# Diabetes exacerbates destructive inflammation by activating the CD137L-CD137 axis in dendritic and IL-17^+^ T cells

**DOI:** 10.1172/JCI193289

**Published:** 2025-12-11

**Authors:** Xin Huang, Min Liu, Michael V. Gonzalez, Rahul Debnath, Hamideh Afzali, Yongwon Choi, Su Ah Kim, Kang I. Ko, Dana T. Graves

**Affiliations:** 1 Hospital of Stomatology, Guanghua School of Stomatology and; 2 Guangdong Provincial Key Laboratory of Stomatology, Sun Yat-sen University, Guangzhou, Guangdong, China.; 3 Department of Periodontics, School of Dental Medicine, University of Pennsylvania, Philadelphia, Pennsylvania, USA.; 4 Center for Cytokine Storm Treatment & Laboratory and; 5 Department of Pathology and Laboratory Medicine, Perelman School of Medicine, University of Pennsylvania, Philadelphia, Pennsylvania, USA.

**Keywords:** Immunology, Inflammation, Dendritic cells, Innate immunity

## Abstract

Periodontal disease, a bacterial infection affecting a large percentage of the world’s population, is an important risk factor for several systemic diseases and is significantly worsened by diabetes. To investigate how diabetes exacerbates the inflammatory response to bacteria in this disease, we combined insights from murine and human studies. Through single-cell RNA-Seq, we identified a compelling hyperglycemia-driven molecular pathway: the upregulation of CD137L in dendritic cells (DCs) and increased expression of its receptor, CD137, in IL-17^+^ T cells. The CD137L-CD137 axis emerged as a pivotal mediator of diabetes-induced inflammatory tissue destruction. Antibody-mediated inhibition of CD137L markedly reduced diabetes-driven bone loss, neutrophil recruitment, expansion of γδ T cells, and excessive infiltration by IL-17A^+^ cells. In vitro studies further validated these findings and established that dysregulation of DCs mediated by high glucose levels dramatically altered γδ T cell activity in co-culture systems via CD137L. The essential role of DCs as CD137L producers in vivo was definitively established through lineage-specific Akt1 deletion, which abrogated CD137L expression in DCs and reversed the adverse effects of hyperglycemia on increased IL-17^+^ T cells and loss of Tregs in vivo. Conversely, activation of CD137 with an agonist in normal animals recapitulated diabetes-induced abnormalities in the inflammatory response and accelerated bone loss. These findings elucidate a key mechanism underlying diabetes-induced immune dysregulation and inflammatory damage, and point to the CD137L-CD137 pathway as a promising therapeutic target, offering potential insights into mitigating other diabetes-associated complications linked to inflammatory changes.

## Abstract 2

Diabetes worsens inflammatory damage induced by bacterial challenge by stimulating dendritic cell-produced CD137L, which activates γδ T-cells through CD137, leading to a dysregulated host response.

## Introduction

Periodontal disease constitutes a substantial global health problem, affecting a substantial proportion of the world’s population annually (1). It is triggered by bacteria-induced inflammation of the periodontal tissues surrounding the teeth and is one of the most common inflammatory diseases worldwide (1, 2). The etiological process involves the formation of a bacterial dysbiosis in the oral cavity that disrupts immune homeostasis in the adjacent gingiva, leading to greater inflammation and subsequent production of enzymes that break down gingival connective tissues attached to the tooth surface and release of factors that stimulate osteoclast-mediated bone resorption (3, 4). The inflammatory response observed in both human and animal models involves cells of the innate and adaptive immune response that are present simultaneously and interact to amplify periodontal inflammation, connective tissue destruction, and bone loss (3, 4). Clinically, periodontitis can lead to substantial tooth loss and reduction in quality of life. The implications of periodontal disease extend beyond oral health because it is linked to several systemic conditions, including cardiovascular diseases, diabetes mellitus, Alzheimer’s disease, rheumatoid arthritis, and some forms of cancer (5, 6). For many of these associations, there is evidence of a causal relationship linked to the systemic dissemination of oral bacteria (7).

One of the strongest links between periodontal disease and systemic condition is diabetes mellitus (7). Numerous clinical trials have established that diabetes significantly worsens periodontal disease, and periodontal disease negatively affects glycemic control (7, 8). The American Academy of Periodontology and the European Federation of Periodontology jointly support that both type 1 and type 2 diabetes increase the severity of periodontal disease (9). Hyperglycemia is a common outcome of both type 1 and 2 diabetes and can lead to increased chronic inflammation and several diabetic complications (7–10). The inflammatory burden enhanced by diabetes has a dramatic impact on periodontitis in part due to an increase in the pathogenicity of the oral microbiome and greater inflammation-induced bone loss (7, 10, 11).The inflammatory changes are credited to changes in neutrophil function, reduced conversion from a pro-inflammatory M1 macrophage phenotype to an M2 phenotype (12, 13), the presence of advanced glycation end products (7), and an increased expression of pro-inflammatory cytokines (4, 7). Despite these advances, there is an incomplete understanding of how diabetes alters the cellular subpopulations, their respective states, and the pathways through which diabetes exacerbates periodontitis. As a result, there is an important need to elucidate cell-specific dynamics that are modulated by diabetic conditions and to develop a comprehensive functional annotation of cell activity.

New advances in single-cell transcriptomics have emerged that provide an exponential improvement in quantifying the behavior of specific cell types in various disease states. Through the use of bioinformatics, single-cell RNA-Seq (scRNA-Seq) has provided a greater understanding of how epithelial cells and fibroblasts participate in the response to bacterial challenge in periodontitis (14–18). These studies suggest that specific ligand-receptor pairs are crucial in driving inflammation in response to bacterial dysbiosis, although many have yet to be functionally verified (14, 18, 19). Additionally, single-cell studies have shown that leukocytes, including macrophages and DCs, can exhibit various proinflammatory subsets associated with cytokine and chemokine production, which potentially modulate immune cell interactions and bone remodeling during bacterial challenge in periodontitis (16, 20).

RAC-α serine/threonine kinase, also known as AKT1, is a key downstream effector of PI3K signaling and plays a central role in cellular metabolism, survival, and immune regulation (21). Akt activity in DCs increases T cell activation and inflammation (22, 23), and it has an anti-apoptotic function that is linked to increased immune cell survival and a greater inflammatory response (24). Given that excessive inflammation and T lymphocytes are thought to play key roles in periodontal disease and diabetes enhances periodontal inflammation, we hypothesized that AKT1 in DCs might mediate diabetes-exacerbated periodontal inflammation by influencing downstream immune interactions.

We used scRNA-Seq to create an atlas of the inflammatory-cell changes modulated by diabetes in the gingiva and undertook mechanistic studies based on these results. Diabetes significantly increased bone loss that was associated with changes in DCs and alterations in γδ T cells and Tregs. Surprisingly, lineage-specific deletion of Akt1 in DCs completely reversed diabetes-enhanced bone loss, diabetes-increased IL-17^+^ cells and diabetes-induced loss of Tregs. scRNA-Seq analysis identified dysregulation of the CD137L-CD137 axis as a key downstream effector of Akt1 in DCs that was confirmed through in vivo and in vitro experiments. CD137 (also known as 4-1BB) is a costimulatory receptor expressed on activated T cells and other immune cells (25, 26), whereas CD137L (also known as 4-1BBL and TNFSF9) is expressed on antigen-presenting cells such as DCs (27, 28). This ligand-receptor interaction plays a key role in T cell activation, survival, and effector function and has been implicated in the amplification of inflammation in infectious and autoimmune diseases. However, whether this pathway contributes to enhanced immune dysregulation under diabetic conditions has not been previously explored.

Blockage of CD137L rescued diabetes-enhanced inflammation and bone loss triggered by application of periodontal pathogens; exogenous activation of CD137 in vivo mimicked the effect of diabetes on exacerbating periodontitis. These findings unveil what we believe to be a novel mechanistic role for the CD137L (TNFSF9) – CD137 (TNFRSF9) axis in orchestrating a diabetes-amplified inflammatory cascade initiated by bacterial challenge. This axis drives a deleterious pathophysiological process, wherein diabetes causes dysregulation of this axis to exacerbate tissue-destructive inflammation.

## Results

### Dendritic cells play a key role in diabetes-enhanced periodontitis.

**Figure 1:**
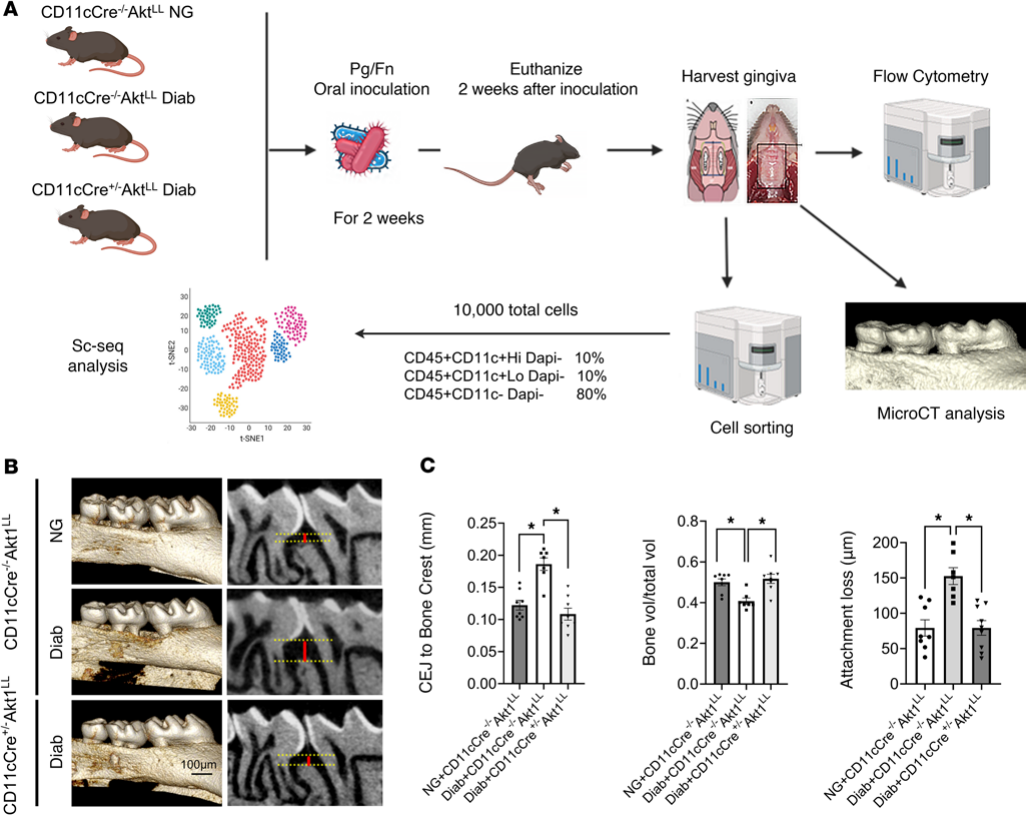
DC-specific AKT1 deletion reverses diabetes-induced periodontal bone loss. (**A**) Schematic diagram of the experimental design and single cell RNA-Seq (scRNA-Seq) workflow. Fn, *Fusobacterium nucleatum*; Pg, *Porphyromonas gingivalis*. (**B**) Representative microCT images of the 3D reconstruction of the maxillary molar teeth in NG CD11c.Cre^–/–^.Akt1^LL^, diabetic CD11c.Cre^–/–^.Akt1^LL^, and diabetic CD11c.Cre^+/–^.Akt1^LL^ groups. Yellow lines mark the CEJ and alveolar bone crest (ABC); red lines represent the distance from the CEJ to ABC. (**C**) microCT analysis of periodontitis by measuring the distance between the CEJ and alveolar bone crest (left panel). The amount of bone was also quantified as the percentage of bone in the interdental space between the first and second maxillary molar teeth divided by the total area of this space (middle panel). Quantitation of attachment loss by measuring the distance from the CEJ to the base of the epithelial attachment through histomorphometric analysis (right panel). *n* = 7–9 animals/group. Statistical analysis was performed using a 1-way ANOVA followed by Tukey’s post hoc test. *P < 0.05. Vol, volume.

**Figure 2:**
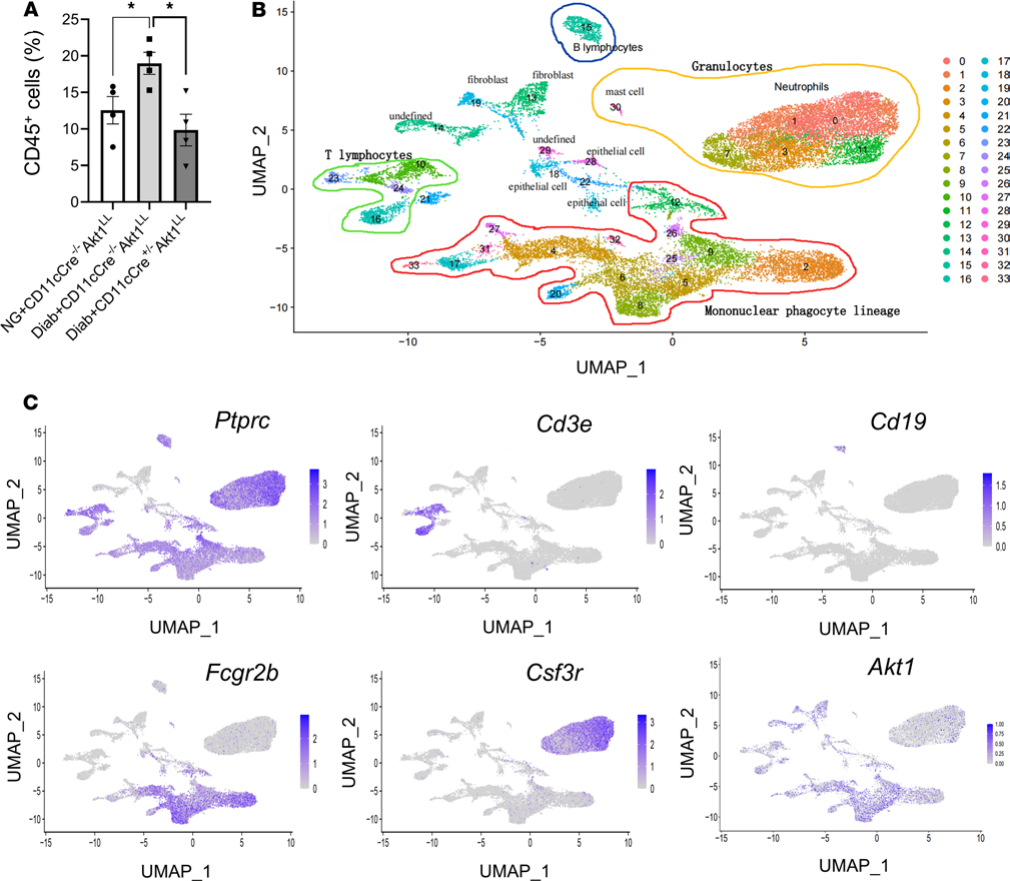
Single-cell profiling of gingival leukocytes. Periodontitis was induced and cells were isolated from gingival tissue as described in Figure 1A, and flow cytometry or FACS sorting to enrich leukocytes was carried out as described in Figure 1A. (**A**) The percent of CD45^+^ cells from each group was determined by flow cytometry. Data are expressed as the mean ± SEM of 4 independent experiments of cells obtained from gingiva. (**B**) UMAP of scRNA-Seq data from mouse gingiva consisting of 34 clusters. Three primary leukocytes subpopulations are highlighted: granulocytes (yellow-outlined area), mononuclear phagocytes (red-outlined area), and T and B lymphocytes (green- and blue-outlined areas, respectively). (**C**) Feature plots showing the expression of key markers in leukocyte subtypes: *Ptprc* (leukocytes), *Cd3e* (T lymphocytes), *Cd19* (B lymphocytes), *Fcgr2b* (mononuclear phagocytes), *Csf3r* (neutrophils), and the distribution of *Akt1* transcript across clusters in UMAP visualizations. Statistical analysis was performed using a 1-way ANOVA followed by Tukey’s post hoc test. *P < 0.05.

Previous studies suggested that DCs play a role in periodontitis (29, 30). We carried out studies in a well-established mouse model in which periodontitis was induced by oral inoculation of the periodontal pathogens *Porphyromonas gingivalis* and *Fusobacterium nucleatum* (31, 32); diabetes was induced by multiple low-dose injections of streptozotocin (STZ). To investigate the role of DCs in diabetes-enhanced periodontitis, we examined normoglycemic (NG) WT mice (NG CD11c.Cre^–/–^.AKT1^LL^), diabetic WT mice (Diab WT) (Diab CD11c.Cre^–/–^.AKT1^LL^) and diabetic mice with lineage-specific deletion of Akt1 in DCs (Diab CD11c.Cre^+/–^.AKT1^LL^) (Figure 1A), with the experimental and control groups consisting of littermates. microCT results showed that diabetic mice had a 52% increase (*P* < 0.05) in the distance from the cementoenamel junction (CEJ) to the alveolar crest and a significant (*P* < 0.05) 23% reduction in the interdental alveolar bone area when compared with NG WT mice, both of which were restored with Akt1 deletion in DCs (*P* < 0.05) (Figure 1, B and C). Histomorphometric analysis demonstrated that compared with the NG WT group, Diab WT mice had significantly increased periodontal tissue damage with a 93% increase (*P* < 0.05) in connective tissue attachment loss as measured by the distance from the CEJ to the base of the epithelial attachment (Figure 1C and Supplemental Figure 1, A–C; supplemental material available online with this article; https://doi.org/10.1172/JCI193289DS1). These parameters were rescued by Akt1 deletion in DCs in the diabetic group (*P* < 0.05) (Figure 1C and Supplemental Figure 1, A–C), demonstrating the key role of DCs. The infiltration of leukocytes, representing the overall level of inflammation, followed a similar pattern. The CD45^+^ leukocyte population increased significantly with diabetes and was restored to normal levels in diabetic mice with AKT1 deletion in DCs (Figure 2A). These in vivo results establish that DCs have a key role in the formation of inflammatory cell infiltrate and increased periodontal tissue destruction caused by diabetic conditions.

### scRNA-Seq analysis of the immune dysregulation caused by hyperglycemia.

We carried out scRNA-Seq to examine how diabetes alters immune cell behavior in periodontitis and investigated the role of DCs in this process. Cells were obtained from mouse gingiva two weeks after completion of *P*. *gingivalis* and *F*. *nucleatum* oral inoculation to induce periodontitis (33). FACS cell sorting with Abs specific for CD45 and CD11c was carried out to obtain a leukocyte population at an 8:1:1 ratio of CD45^+^CD11c^–^ to CD45^+^CD11c^high^ to CD45^+^CD11c^low^ to obtain leukocytes and enrich the dendritic CD11c^high^ and CD11c^low^ populations. R/Seurat thresholds were set at 200 unique molecular identifier(s) (UMIs) to exclude cells with low UMI counts, at 2,500 UMI to filter out potential doublets, and at 10% mitochondrial gene transcripts to remove likely apoptotic cells. After filtering, the Seurat suite of tools was used to normalize, scale, and cluster 21,450 cells into 34 clusters, as displayed in the uniform manifold approximation and projection (UMAP) (Figure 2B) (34). Mononuclear phagocyte lineage cells were identified in clusters 2, 5, 6, 8, 9, 12, 17, 20, 25, 26, 27, 31, and 33 and were shown to express the canonical marker genes *Ptprc* (*CD45*) and *Fcgr2b*, consistent with monocytes, macrophages, and DCs (Figure 2C). T cells were found in clusters 10, 16, 23, and 24, marked by *Ptprc* and *Cd3e* transcripts (Figure 2C). B lymphocytes were located in cluster 15, identified by *Ptprc* and *Cd19* (Figure 2C). Granulocytes were present in clusters 0, 1, 3, 7, 11, and 30, marked by *Ptprc* and *Csf3r* transcripts (Figure 2C). Even though FACS sorting was carried out, some relatively small clusters of cells were obtained that did not express *Ptprc*, including clusters 13 and 19 (fibroblasts); clusters 18, 22, and 28 (epithelial cells); and 14, 21, and 29 (unidentified), which were excluded from further analysis (Figure 2C). The assignments of clusters to cell types and the proportion that each represented in the total cell population are presented in Table 1.

We also investigated whether lineage-specific AKT1 deletion in CD11c^+^ cells affects the systemic population of DCs, T cells, and B cells. Splenocytes were isolated from experimental mice (CD11c.Cre^+/–^.AKT1^LL^) and the control littermates (CD11c.Cre^–/–^.AKT1^LL^) and immunostained for DCs, T cells, and B cells by flow cytometry. The data revealed no significant differences in the systemic levels of DC, T cell, and B cell populations between experimental and control littermates (data not shown).

**Figure 3:**
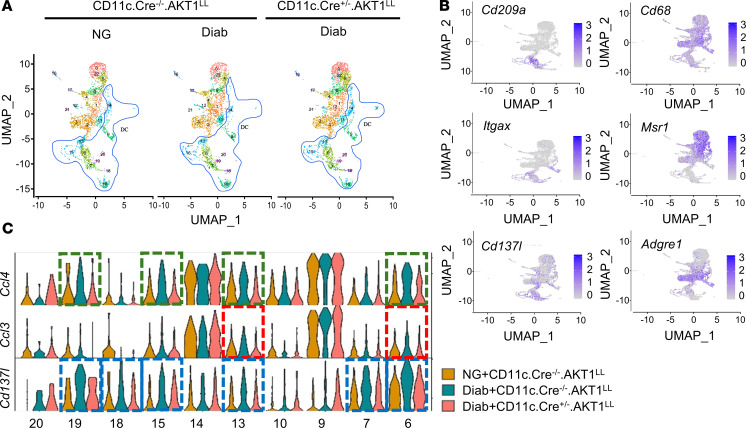
Diabetes-alters *Ccl4*, *Ccl3*, and *Cd137l* transcripts in gingival DCs. Periodontitis was induced and cells were isolated from gingival tissue as described in Figure 1A. After FACS sorting to enrich leukocytes, scRNA-Seq was performed. (**A**) UMAP visualization of mononuclear phagocyte subclusters of cells from NG CD11c.Cre^–/–^.Akt1^LL^, diabetic CD11c.Cre^–/–^.Akt1^LL^, and diabetic CD11c.Cre^+/–^.Akt1^LL^ mice after initial clustering described in Figure 2. Blue lines encircle DC subclusters. (**B**) Feature plots displaying the expression of markers in mononuclear phagocyte subtypes: *Cd209a* and *Itgax* (DC); *Cd68, Msr1*, and *Adgre1* (macrophages); and *Cd137l* transcripts. (**C**) Violin plots showing *Ccl4, Ccl3*, and *Cd137l* transcript levels in individual DC subclusters.

To analyze the cell populations in more detail, we divided the gingiva mucosal cells in Figure 2B into 3 major populations and then rescaled and reclustered them within each population using R/Seurat. The mononuclear phagocytes were reclustered into 24 distinct subpopulations (Figure 3A). Ten subclusters of DCs (6, 7, 9, 10, 13, 14, 15, 18, 19, and 20) were identified; they were marked by the expression of canonical marker genes *Itgax* and *CD209a* (Figure 3B). Among these, subclusters 9, 14, and 19 were plasmacytoid DCs. Monocyte/macrophage subclusters, including subclusters 0, 1, 2, 3, 4, 5, 8, 11, 12, 16, 21, and 22, were identified by the expression of marker genes *Cd68*, *Msr1*, and *Adgre1* (Figure 3B). The percentages of total DCs and total macrophages were similar among the experimental and control groups examined: NG WT, Diab WT, and Diab CD11c.Cre^+/–^.AKT1^L/L^ with Akt1 deletion in DCs (Table 2).

To better understand the impact of diabetes on these cells, we focused on the differentially expressed genes that were upregulated by diabetes and whose upregulation was reversed by lineage specific Akt1 deletion in DCs. The thresholds we used were adjusted to *P* values <0.1 and log2 fold change (log2FC) increases greater than 0.25, as shown in Table 3. Among these genes, *Cd137l (Tnfsf9*), *Ccl3*, and *Ccl4* attracted our attention because they met these thresholds and also are known to play a key role in inflammatory responses. The *Cd137l*^+^ cells were strongly associated with the DC marker *Itgax*, as shown in the mononuclear phagocyte feature plots (Figure 3B); however, the cellular linkage of *Ccl4*^+^ cells was not as specific. Table 4 shows the FindAllMarkers analysis (in Seurat), which demonstrated that the cells with the highest level of *Cd137l* expression, as reflected by log2FC expression and percentage of cells expressing *Cd137l* (pct 1) were DCs. The only other cell cluster that expressed *Cd137l* was a macrophage cluster (subcluster 2). Violin plots identified upregulation of *Cd137l* in DC clusters 6, 7, 13, 15, 18, and 19 from the Diab WT group compared with the NG WT group (Figure 3C). Interestingly, DC clusters 6 and 7 had a significant increase in *Cd137l*, which was reversed by lineage-specific deletion of AKT1 in diabetic mice but not in macrophage cluster 2 (Supplemental Table 3). *Ccl4* in subclusters 6, 13, 15, and 19, and *Ccl3* in subclusters 6 and 13 were upregulated by diabetes and the upregulation was reduced by lineage-specific deletion of Akt1 in DCs of diabetic mice, as shown in Figure 3C.

**Figure 4:**
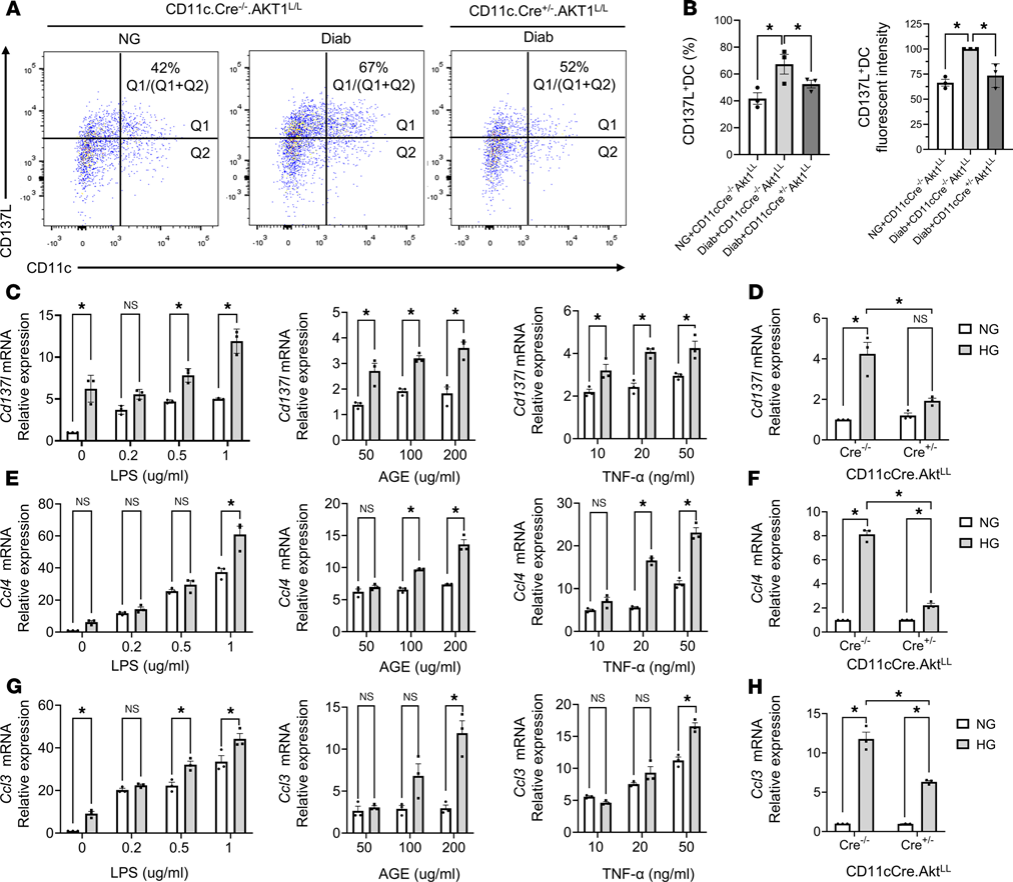
Modulation of *Ccl4, Ccl3*, and *Cd137l* levels in DCs. (**A**) Representative flow cytometry plots of CD11c and CD137L expression in cells isolated from gingival tissue as described in Figure 1A. Cells were pregated for live CD45^+^ leukocytes. (**B**) Quantitative analysis of CD137L^+^CD11c^+^ cells normalized per total CD11c^+^ cells (left) and mean fluorescent intensity of CD137L in CD11c^+^ cells (right). Data are expressed as the mean ± SEM of 3 independent experiments. (**C**) RNA was extracted from bone marrow–derived WT DCs cultured in NG or HG medium without or with LPS, AGE, or TNF stimulation and examined by RT-qPCR. (**D**) Quantitation of *Cd137l* mRNA in DCs isolated from CD11c.Cre^–/–^.Akt1^L/L^ or CD11c.Cre^+/–^.Akt1^L/L^ mice incubated in NG or HG medium. (**E**) Cells from (**C**) were examined for *Ccl4* mRNA in DCs. (**F**) Cells from(**D**) were examined for *Ccl4* mRNA in DCs. (**G**) Cells from (**C**) were examined for *Ccl3* mRNA in DCs. (**H**) Cells from (**D**) were examined for *Ccl3* mRNA in DCs. Statistical analysis was performed using a 1-way ANOVA followed by Tukey’s post hoc test. (**C**, **E**, and **G**) Data are expressed as the mean ± SEM of 3 independent experiments (*n* = 3). Comparisons between NG and HG groups were conducted using Student’s *t* test. (**D**, **F**, and **H**) Each value represents the mean of 3 biological replicates (*n* = 3), analyzed using 1-way ANOVA and Tukey’s post hoc test. *P < 0.05.

Validation experiments were performed using flow cytometry to identify changes in CD45^+^CD11c^+^CD137L^+^ DCs. Diabetes increased CD45^+^CD11c^+^CD137L^+^ DCs by 62% compared with NG mice, and this increase was largely rescued by Akt1 deletion in DCs (*P* < 0.05) (Figure 4, A and B), with the gating justified by fluorescence minus one (FMO) control (Supplemental Figure 2, C and D). Flow cytometry was also used to examine CD137L expression by fluorescence intensity, which increased by 60% in the Diab WT mice compared with the matched NG WT mice (*P* < 0.05) (Figure 4B). The increase was largely rescued in diabetic mice with AT1 deletion in DC (*P* < 0.05) (Figure 4B). Thus, diabetes significantly increased the number of DCs expressing CD137L and the intensity of expression per cell, which were *Akt1* dependent.

To determine if the impact of diabetes on DCs was dependent on a high-glucose (HG) environment, we examined *Cd137l* expression in bone marrow–derived DCs (BMDCs) incubated in normal or HG medium in vitro (Figure 4, C–H). To simulate inflammatory conditions present in diabetic periodontitis, DCs were also challenged with bacterial LPS, an advanced glycation end product (AGE) carboxymethyllysine-modified BSA, or TNF. Under NG conditions, *Cd137l* expression stimulated by LPS in DCs was less than that stimulated by LPS in HG (Figure 4C). Similarly, HG increased the *Cd137l* expression in DCs in response to AGE or TNF, up to a 2.0-fold increase for both (*P* < 0.05) (Figure 4C), indicating that the effect of diabetes on increased *Cd137l* levels can be tied directly to HG levels.

To extend results obtained in vivo, we determined if HG-upregulated *Cd137l* expression was Akt1 dependent, using DCs from NG WT CD11c.Cre^–/–^.Akt1^LL^ and NG experimental CD11c.Cre^+/–^.Akt1^LL^ mice. Although the HG condition resulted in significantly increased *Cd137l* expression in DCs isolated from control WT CD11c.Cre^–/–^.Akt1^LL^ mice, this effect was dramatically reduced in DCs with Akt1 deletion from CD11c.Cre^+/–^.Akt1^LL^ experimental mice, comparable to normal levels (*P* < 0.05) (Figure 4D). We also determined if HG altered chemokine expression. The HG condition enhanced the levels of *Ccl4* level 2.0-fold in DCs compared with incubation with 1 μg/mL LPS in NG conditions (*P* < 0.05) (Figure 4E). We also observed a similar 2- to 3-fold increase in *Ccl4* levels in DCs exposed to AGE or TNF in HG compared with NG medium (*P* < 0.05) (Figure 4E). The impact of HG on *Ccl4* was prevented in DCs isolated from experimental CD11c.Cre^+/–^.Akt1^L/L^ mice (*P* < 0.05, Figure 4F). Similar results were observed with *Ccl3* (*P* < 0.05) (Figure 4, G and H). Thus, the response of DCs to mediators that stimulate inflammation was significantly increased by exposure of DCs to the HG condition.

### Impact of hyperglycemia and DCs on γδ T cells.

**Figure 5:**
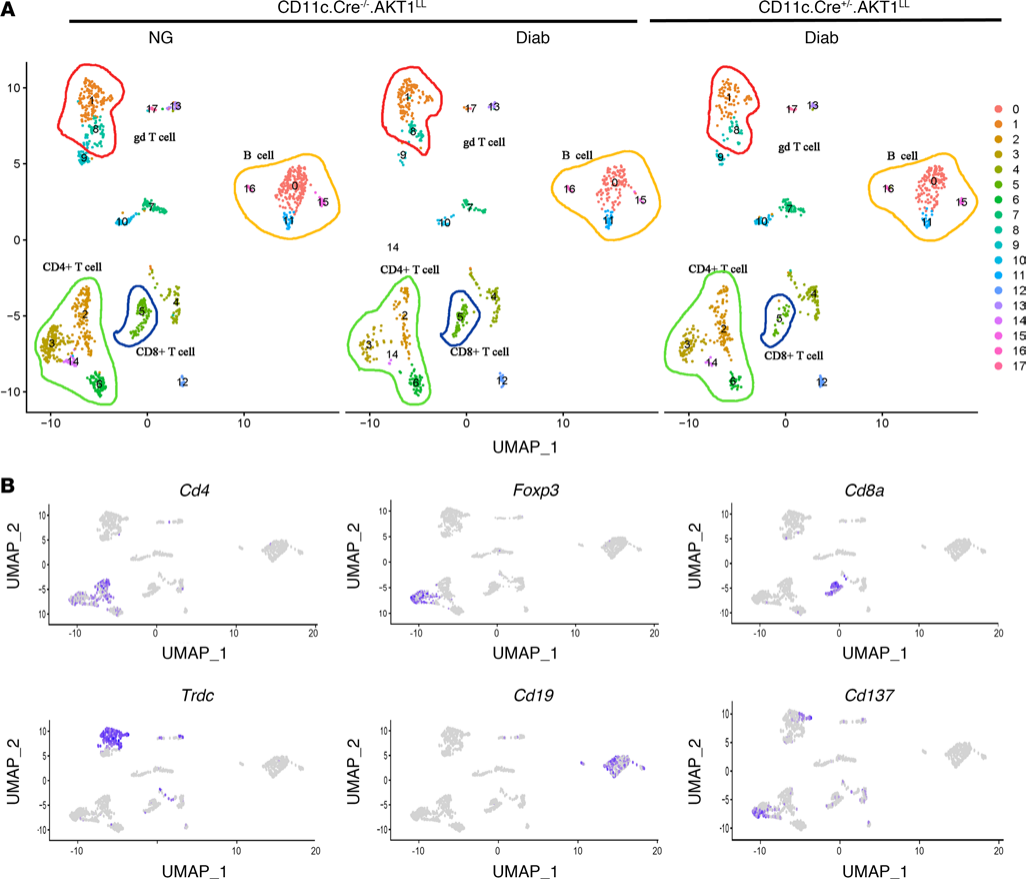
scRNA-Seq profile of gingival lymphocyte subclusters. Periodontitis was induced and cells were isolated from gingival tissue as described in Figure 1A. After FACS sorting to enrich leukocytes, scRNA-Seq was performed. (**A**) UMAP visualization of lymphocyte subclusters from NG CD11c.Cre^–/–^.Akt1^LL^, diabetic CD11c.Cre^–/–^.Akt1^LL^, and diabetic CD11c.Cre^+/–^.Akt1^LL^ mice from initial clustering described in Figure 2. Yellow outline highlights B cell subclusters; green outline highlights CD4^+^ T cell subclusters; blue circle outlines CD8^+^ T cell subclusters; red circle outlines γδ T cell subclusters. (**B**) Feature plots displaying *Cd4* (CD4^+^ T cells), *Foxp3* (Tregs), *Cd8a* (CD8^+^ T cells), *Trdc* (γδ T cells), *Cd19* (B cells), and *Cd137* transcripts.

Subclustering of lymphocytes was performed by R/Seurat based on the initial clustering shown in Figure 2B for a more refined analysis based on *Cd3* transcripts. The lymphocytes were classified into 18 distinct subpopulations (Figure 5A). They include 4 subclusters of *Cd4* expressing T cells (subclusters 2, 3, 6, and 14), 1 cluster of T cells marked by *Cd8a* (subcluster 5), and 2 clusters of γδ T cells marked by *Trdc* (subclusters 1 and 8) (Figure 5B). Within the CD4^+^ T cell population, a Treg cluster marked by *Foxp3* was identified in subcluster 3. Additionally, 4 subclusters of B cells were identified, marked by the expression of the canonical marker gene *Cd19* (subclusters 0, 11, 15, and 16) (Figure 5B).

**Figure 6:**
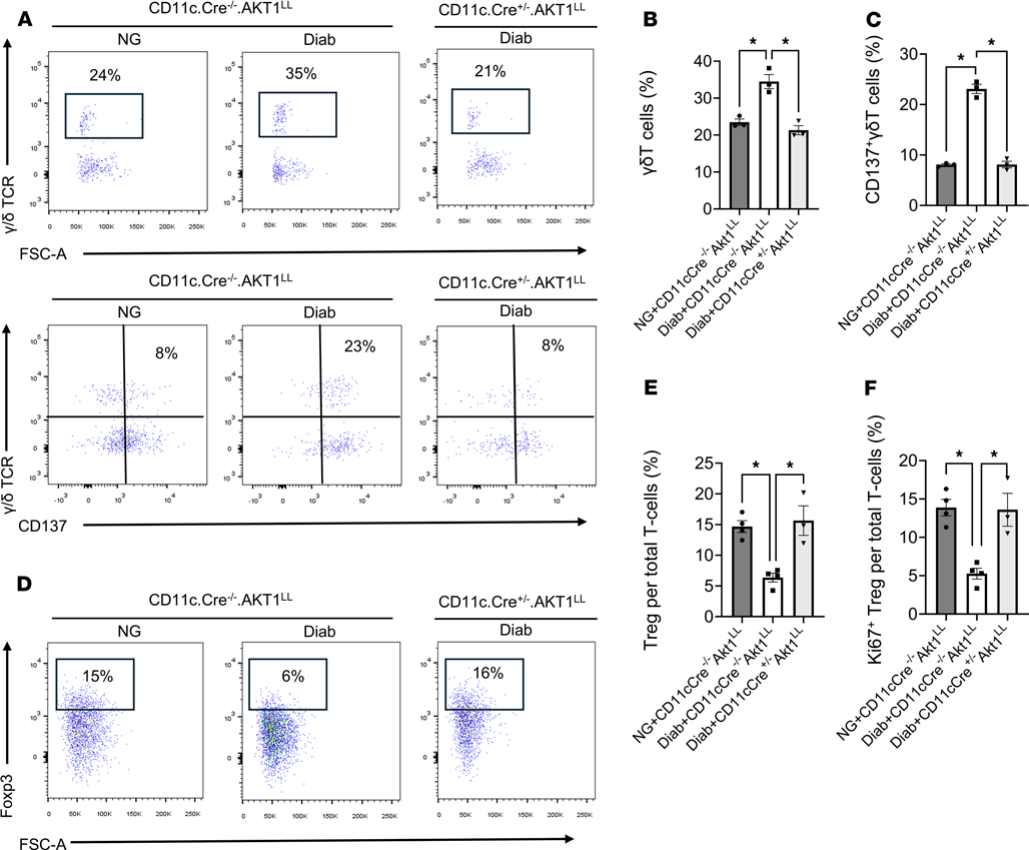
Diabetes increases γδ T cells and CD137^+^ γδ T cells that are DC dependent. Periodontitis was induced and flow cytometry was carried out on cells isolated from murine gingival tissue as described in Figure 1A. (**A**) Representative flow cytometry plots for γδTCR^+^ cells (top row) and γδTCR^+^CD137^+^ cells (bottom row). Cells were pregated for live CD45^+^CD3^+^ cells. (**B** and **C**) Quantitation of the percent γδ T cells per total T cells and the percent CD137^+^γδ T cells per total T cells. (**D**) Representative flow cytometry plots of Foxp3^+^ T cells per total T cells in mouse gingival cells after gating for live T lymphocytes (CD45^+^CD3^+^). (**E** and **F**) Quantitation of Foxp3^+^ T cells per total T cells and Ki67^+^ Tregs per total T cells. (**B**, **C**, **E,** and **F**) *n* = 3–4 biological replicates per group. *P < 0.05, 1-way ANOVA with Tukey’s post hoc test.

scRNA-Seq data indicated that γδ T cells increased by 35% in the Diab WT group compared with the NG WT group (Table 2). This increase was rescued in diabetic mice with AKT1 deletion in DCs, indicating that Akt1 in DCs has a pronounced effect on γδ T cells. In contrast, the effect of diabetes on CD4^+^ T cells was the opposite, being 42% higher in the NG WT mice compared with the diabetic WT mice, which was partially restored in diabetic mice with AKT1 deletion in DCs. (Table 2). *CD137*, the receptor for CD137L, was expressed by γδ T cells (lymphocyte cluster 1), Tregs (lymphocyte cluster 3), and NK T cells (lymphocyte clusters 4 and 12) (Figure 5B and Table 5). The other clusters had low levels of *CD137* expression, as reflected by the percentage of positive cells and the low average log2FC values. Flow cytometry validated that γδ T cells were increased by 47% in the Diab WT group compared with the matched NG WT group, which was reversed in Akt1-deleted DCs from the diabetic group (*P* < 0.05) (Figure 6, A and B). The change in Tregs assessed by flow cytometry was also consistent with the scRNA-Seq data. Tregs were significantly reduced in the Diab WT group compared with the NG WT group (*P* < 0.05) and rescued by Akt1 deletion in DCs from the diabetic group (*P* < 0.05) (Figure 6, D and E, and Table 2). These results point to the impact of hyperglycemia on DCs as a driving force mediating the effect of diabetes on shifts in lymphocyte populations. Interestingly, diabetes also affected expression of CD137, the receptor for CD137L, in lymphocytes. Diabetes increased by approximately 3-fold the percentage of γδ T cells that expressed CD137; the percentage returned to normal levels in the diabetic group when Akt1 was deleted in DCs (Figure 6, A and C). Taken together, scRNA-Seq analysis and flow cytometry data indicate that diabetes increases the expression of CD137L in DCs and the number of γδ T cells expressing CD137, suggesting a mechanism whereby diabetes enhances expression of both components of the CD137L-CD137 axis, both of which are dependent on Akt1 in DCs. This is noteworthy because the CD137L-CD137 axis drives the proliferation of T cells, including γδ T cells. In contrast, the percentage of Tregs significantly decreased in the Diab WT group; this decrease was rescued by Akt1 deletion in DCs in vivo, and the percentage of Tregs expressing Ki67 in vivo (Figure 6, D and E) was also reduced by diabetes and restored by Akt1 ablation in DCs in diabetic mice (*P* < 0.05) (Figure 6F).

**Figure 7:**
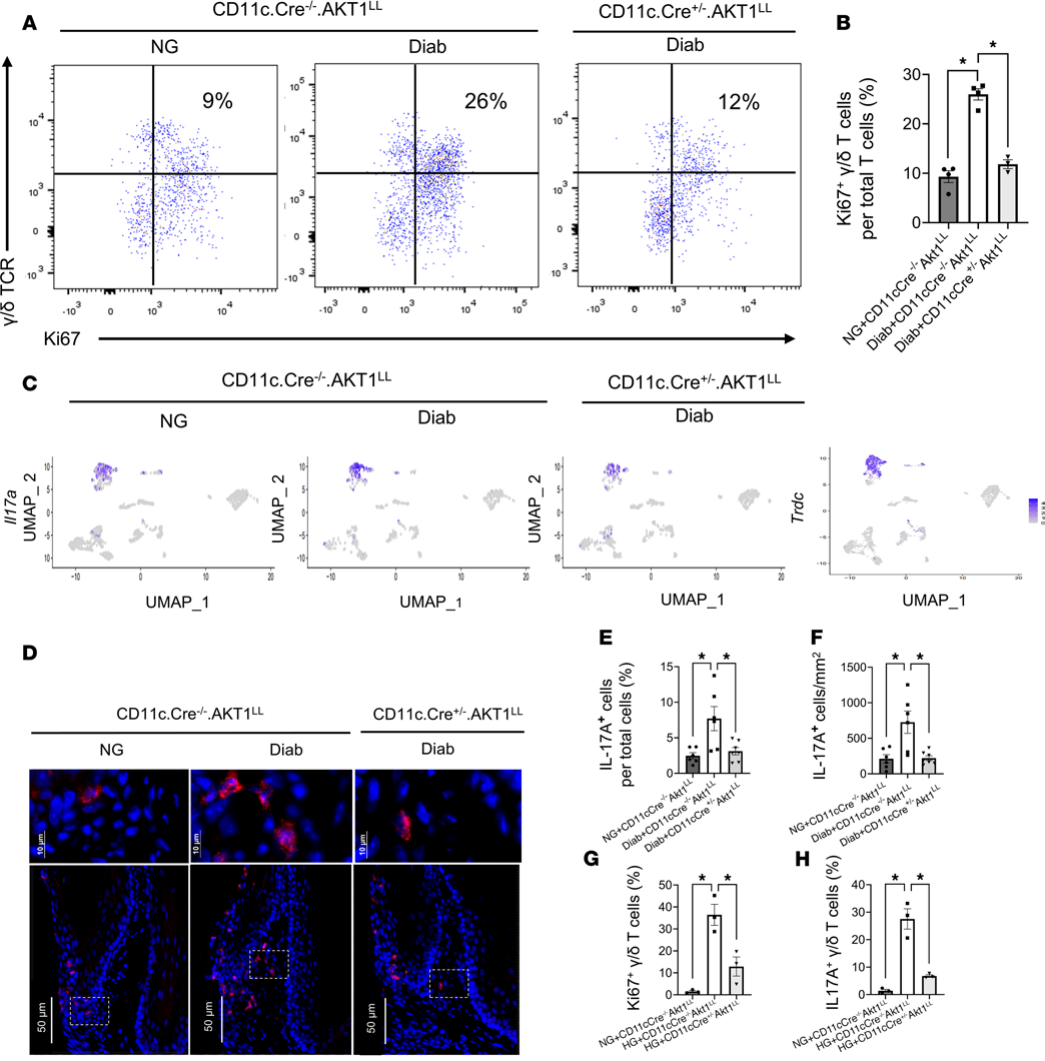
Diabetes increases proliferation and IL7A expression in γδ T cells that are DC dependent. Periodontitis was induced and flow cytometry was carried out on cells isolated from murine gingival tissue as described in Figure 1A. (**A**) Representative flow cytometry plots of γδ TCR and Ki67 expression in mouse gingival cells isolated from NG CD11c.Cre^–/–^.Akt1^LL^, diabetic CD11c.Cre^–/–^.Akt1^LL^, and diabetic CD11c.Cre^+/–^.Akt1^LL^ mice. (**B**) Quantitation of proliferating γδ T cell (Ki67^+^γδTCR^+^) per total T cells. *n* = 3–4 biological replicates per group. *P < 0.05, 1-way ANOVA with Tukey’s post hoc test. (**C**) Lymphocyte subclusters shown in Figure 5 were examined by UMAP visualization for *IL-17a* transcripts and localized to a *Trdc^+^* γδ T cell subpopulation. (**D**) Representative immunofluorescence images of IL-17A (red) expression in gingival tissues. Inset images show IL-17A^+^ cells in the connective tissue compartment. (**E** and **F**) Quantitation of IL-17A^+^ cells from (**D**) normalized per the number of total cells (**E**) and IL-17A^+^ cells per connective tissue area (**F**) for 3 mouse groups. (**G** and **H**). Enriched populations of DCs and gδ T cells were co-cultured in vitro in media with NG levels or 25 mM HG and then examined by flow cytometry. (**G**) Quantitation of Ki67^+^ γδ T cells normalized per total γδTCR^+^ cells from CD11c.Cre^–/–^.Akt1^L/L^ or CD11c.Cre^+/–^.Akt1^L/L^ mice. (**H**) Quantitation of IL-17A^+^ γδ T cells normalized per total γδTCR^+^ cells. (**E** and **F**) *n* = 6–7 biological replicates per group. (**G** and **H**) *n* = 3 biological replicates per group. *P < 0.05, 1-way ANOVA with Tukey’s post hoc test.

The results from scRNA-Seq and flow cytometry led us to hypothesize that the proliferation of γδ T cells may be enhanced by diabetes through the interaction between CD137L^+^ DCs and γδ T cells. To test this, we examined the expression of the proliferation marker Ki67 in γδ T cells in vivo by flow cytometry analysis. The percentage of Ki67^+^ γδ T cells was approximately 3-fold higher in the Diab WT group compared with the NG WT group (*P* < 0.05), an effect blocked by Akt1 deletion in DCs in diabetic mice (*P* < 0.05) (Figure 7, A and B). To link the increased numbers of γδ T cells to enhanced inflammation and periodontal bone loss, we examined IL-17, a proinflammatory cytokine expressed by the γδ T cells in periodontitis (35). *IL-17a* expression in lymphocytes clusters is shown in Table 6 using the FindAllMarkers function in R/Seraut to identify *IL-17a*-expressing cells. The only cells with positive average log2FC for *IL-17a* were γδ T cells and 54% to 86% of the γδ T cells in these clusters expressed *IL-17a*. In cluster 1, *IL-17a* was increased in the Diab WT group compared with the NG WT (Table 7). Immunofluorescence with an Ab specific to IL-17A was carried out to confirm that diabetes increased IL-17A expression in NG WT mice that was reversed in diabetic mice with lineage-specific deletion of Akt1 in DCs (Figure 7, C and D). Quantitatively, diabetes increased the IL-17A^+^ cells 3- to 4-fold, which was completely reversed by Akt1 deletion in DCs of diabetic mice. (*P* < 0.05) (Figure 7, E and F).

These in vivo results suggest direct paracrine signaling between CD137L^+^ DCs and CD137^+^ γδ T cells drives γδ T-cell proliferation and IL-17A expression in a diabetic microenvironment. To test this and its dependence on Akt1 in DCs, we carried out co-culture experiments in vitro using purified splenic γδ T cells and DCs isolated from NG WT CD11c.Cre^–/–^.Akt1^L/L^ mice or NG experimental CD11c.Cre^+/–^.Akt1^L/L^ mice (Figure 7, G and H). γδ T cells and DCs were co-cultured at a 4:1 ratio, then exposed to either normal or HG conditions and analyzed by flow cytometry (Figure 7, G and H). In NG medium, γδ T cells exhibited minimal Ki67 expression, which increased more than 20-fold when co-cultured with DCs in HG medium (*P* < 0.05) (Figure 7G). The increase in Ki67^+^ γδ T cells was significantly reduced when co-cultured with Akt1-deleted DCs (Figure 7G). In addition, IL-17A expression in co-cultured γδ T cells exhibited a dramatic 20-fold increase in HG compared with NG conditions, and this increase was blocked when γδ T cells that were co-cultured with Akt-1 deleted DCs (*P* < 0.05) (Figure 7H). These findings indicate that the HG conditions associated with the diabetic microenvironment affect γδ T cell proliferation and IL-17A expression, with DCs playing a key regulatory role in these processes. The pronounced upregulation of IL-17A in diabetic specimens in vivo, coupled with its restoration to baseline levels upon lineage-specific DC-deletion of Akt1 in diabetic mice and the known role of IL-17A in promoting periodontal tissue destruction, point to IL-17A as an important pathogenic end point resulting from the altered crosstalk between DCs and γδ T cells.

### The CD137L-CD137 axis plays a key role in diabetes-altered inflammation and periodontal bone loss.

**Figure 8:**
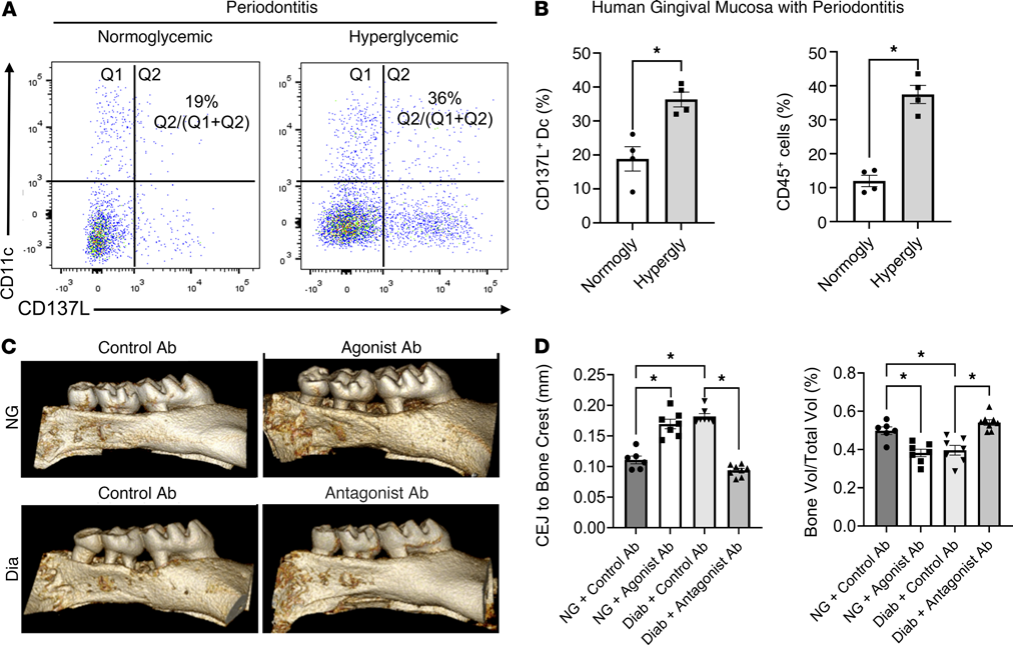
The CD137L-CD137 axis plays a key role in periodontitis severity in hyperglycemic humans and mice. (**A** and **B**) Human gingival specimens were obtained from NG (Normogly) individuals and individuals with hyperglycemia (Hypergly), and cells were isolated for flow cytometry. (**A**) Representative flow cytometry dot plots of CD137L**^+^** and CD11c**^+^** cells from human gingival specimens (*n* = 4). Cells were pregated for live mononuclear phagocytes (Zombie**^–^**CD45^+^HLA-DR^+^). (**B**) Quantitation of CD137L^+^CD11c^+^ DCs per total HLA-DR^+^ mononuclear phagocytes (left) and CD45^+^ leukocytes per total live cells (Zombie**^–^**) in human gingival specimens. (**C** and **D**) Diabetic mice were injected with a CD137L-antagonist Ab or control Ab, and NG mice were injected with a CD137-agonist Ab or control Ab. Periodontitis was induced as described in Figure 1. (**C**) Representative microCT images of 3D reconstruction of the molar teeth. (**D**) microCT analysis of bone loss was assessed by measuring the distance from the CEJ to the alveolar crest (left) and ratio of the bone area remaining per total area; *n* = 6–8 biological replicates per group. *P < 0.05, 1-way ANOVA with Tukey’s post hoc test.

To assess whether CD137L is altered by hyperglycemia in humans, we collected samples from NG and individuals with hyperglycemia treated for periodontitis (*n* = 4/group). Flow cytometry was used to identify CD45^+^HLA-DR^+^CD11c^+^CD137L^+^ cells in the gingival mucosa with the gating justified by FMO (Supplemental Figure 6). Individuals with hyperglycemia had almost double the percentage of DCs in the gingiva that express CD137L compared with the NG group (*P* < 0.05) (Figure 8, A and B). The total population of CD45^+^ leukocytes also increased in the gingival mucosa of individuals with hyperglycemia as a percentage of the total population (*P* < 0.05).

To assess the impact of the CD137L-CD137 axis on inflammation-induced bone loss, gain of function in NG and loss of function in hyperglycemic mice were performed. The former was accomplished by using an agonist Ab that binds to CD137 to activate it (36, 37) and the latter with an antagonist Ab to inhibit CD137L (38). microCT analysis revealed that NG mice that received the CD137 agonist had more tissue damage, with a significant 64% increase in the distance between the CEJ to the bone crest and a significant reduction in the remaining bone area (*P* < 0.05) (Figure 8, C and D), mimicking the detrimental impact of diabetes on bone loss. Conversely, treatment with a CD137L antagonist in diabetic mice completely reversed the diabetes-enhanced loss of bone measured by both parameters (Figure 8, C and D) (*P* < 0.05). These results indicate the CD137L-CD137 axis plays a key role in mediating periodontal bone loss enhanced by diabetes.

**Figure 9:**
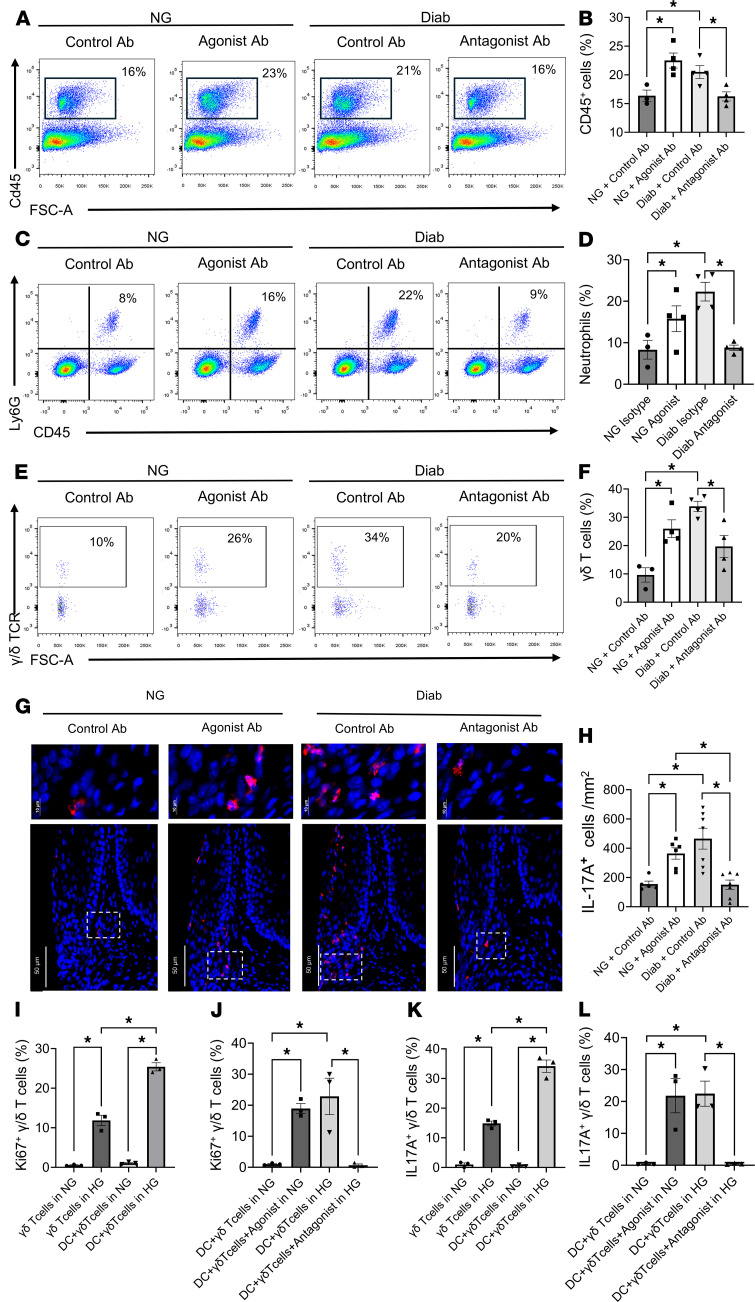
The CD137L-CD137 axis plays a key role in diabetes-altered inflammation. Diabetic mice received a CD137L antagonist or control Ab, and NG mice received a CD137 agonist or control Ab. Periodontitis was induced as in Figure 1. (**A**) Representative flow cytometry plots of CD45^+^ leukocytes from NG+Control Ab, NG+Agonist Ab, Diab+Control Ab, and Diab+Antagonist Ab groups. (**B**) Quantitation of CD45^+^ cells per total live cells. (**C**) Representative flow cytometry plots of Ly6G^+^CD45^+^ neutrophils. (**D**) Quantitation of Ly6G^+^ neutrophils per total CD45^+^ cells. (**E**) Representative flow cytometry plots of γδ T cells pregated as CD45**^+^**CD3**^+^**Zombie**^–^**. (**F**) Quantitation of γδ T cells per total CD3^+^ T cells. (**G**) Representative IL-17A immunofluorescence staining of periodontal tissues, with IL-17A (red) and DAPI (blue) nuclear staining. High-power magnification insets (top row) are identified from lower power images by white boxes. (**H**) Quantitation of IL-17A^+^ cells per mm^2^ of connective tissue area by immunofluorescence. (**I**) For in vitro studies, γδ T cells were cultured alone or co-cultured with DCs under NG or HG conditions. Proliferation of γδ T cells was assessed by Ki67^+^γδTCR^+^ cells. (**J**) Quantitation of proliferating γδ T cells from DC–γδ T cell co-cultures incubated with a CD137 agonist or CD137L antagonist. Cultures without agonist or antagonist Ab were incubated with control Ab. (**K**) Flow cytometric analysis of IL-17A^+^γδ T cells without or with DC co-cultures in NG or HG medium. (**L**) Quantitation of IL-17A^+^γδ T cells in γδ T cell and DC co-cultures incubated with CD137 agonist or CD137L antagonist. Cultures without agonist or antagonist Ab were incubated with control Ab. For in vivo analyses (**B**, **D**, **F**, and **H**), *n* = 3–7 per group; for in vitro assays (**I**–**L**), *n* = 3 per group. Data are presented as mean ± SEM. Statistical significance was determined by 1-way ANOVA with Tukey’s post hoc test. *P < 0.05.

The impact of diabetes and CD137L expression was further examined by flow cytometry after induction of periodontal disease with the gating justified by FMO (Supplemental Figure 7). Treatment of NG mice with a CD137 agonist significantly increased the number of CD45^+^ leukocytes in this group (Figure 9, A and B). Hyperglycemia caused a similar increase in leukocytes, which was blocked in diabetic mice by application of a CD137L antagonist (*P* < 0.05) (Figure 9, A and B). Because neutrophils are thought to contribute to periodontal bone loss, we treated NG mice with the CD137 agonist and examined changes in neutrophil infiltration. The agonist treated group had an almost 2-fold increase in neutrophils (Figure 9, C and D). Diabetes also significantly increased neutrophil infiltration. The increase in neutrophils caused by diabetes was reversed with the CD137L antagonist (Figure 9, C and D), demonstrating the key role of the CD137L-CD137 axis in recruitment of these cells. A similar pattern was observed for γδ T cells, with a significant increase induced by a CD137 agonist and by diabetes, with the latter blocked with a CD137L antagonist (*P* < 0.05) (Figure 9, E and F). These results highlight the role of CD137L in mediating the effect on the leukocyte dysregulation that occurs in hyperglycemic conditions. In addition, treatment with the CD137 agonist in NG mice and the presence of hyperglycemia in diabetic mice dramatically increased IL-17A^+^ T cell counts, whereas the CD137L antagonist reversed this effect in diabetic mice (*P* < 0.05) (Figure 9, G and H). This is noteworthy given our previous results that IL-17A^+^ T cells play a central role in mediating diabetes-enhanced periodontitis (48).

In vitro co-culture experiments were carried out to investigate these results further. HG medium resulted in significantly increased percentage of proliferating (Ki67^+^) γδ T cells compared with NG medium (*P* < 0.05) (Figure 9I). The addition of DCs to γδ T cells in co-cultures increased the percentage of Ki67^+^ γδ T cells by more than 2-fold in HG medium (*P* < 0.05) (Figure 9I). In NG medium, a CD137 agonist significantly increased the percentage of Ki67^+^ γδ T cells (*P* < 0.05) (Figure 9J). Conversely, treatment with a CD137L antagonist completely blocked proliferating γδ T cells in DC - γδ T cell co-cultures in HG medium (*P* < 0.05) (Figure 9J), underscoring the pivotal role of CD137L in mediating the impact of DCs on γδ T cell proliferation in an HG environment. Similarly, γδ T cells co-cultured with DCs had significantly higher IL-17A^+^ expression in response to HG medium compared with γδ T cells alone (*P* < 0.05) (Figure 9K). CD137-agonist treatment significantly enhanced IL-17A^+^ γδ T cells by several fold in NG conditions compared with the IgG control group, mimicking the effect of HG (*P* < 0.05) (Figure 9L). In contrast, CD137L-antagonist treatment reversed the effect of HG on IL-17A^+^ γδ T cells co-cultured with DCs (*P* < 0.05) (Figure 9L). These results, taken together, indicate the CD137L-CD137 communication axis between DCs and γδ T cells is amplified in an HG environment to create expansion of γδ T cells and IL-17A expression. They provide a mechanistic basis for diabetes-induced dysregulation of γδ T cells driven by the CD137L-CD137 axis.

## Discussion

Diabetes mellitus presents significant oral health concerns globally despite insulin treatment. Numerous studies have highlighted the bidirectional relationship between these 2 conditions, because severe periodontitis negatively affects glycemic control, and diabetes worsens periodontal outcomes (39, 40). To investigate the impact of diabetes on periodontal health, we used a well-characterized experimental mouse model and examined human tissues from individuals with periodontitis. The advantage of the mouse model is that inflammation and subsequent tissue loss are predictable and induced by oral administration of the pathogens *P*. *gingivalis* and *F*. *nucleatum*. It is also an excellent model to investigate how diabetes modifies pathology. We focused on DCs, based on previous studies (33, 41), and found that diabetes significantly increases periodontal bone loss that is blocked by DC Akt1 ablation. scRNA-Seq analysis identified significant changes in DCs and γδ T cells caused by diabetes that pointed to the CD137 L-CD137 access. These results were verified in periodontal tissue from human participants with hyperglycemia, and their contribution to the disease pathology aggravated by diabetes was established by the use of function-blocking Abs.

We found through lineage-specific Akt1 deletion that DCs had a dramatic effect in mediating the impact of diabetes on periodontal bone loss. scRNA-Seq analysis established that Akt1 deletion in DCs reversed diabetes-enhanced expression of *Cd137l*, *Ccl4*, and *Ccl3*. These genes play pivotal roles in regulating T cell proliferation and the recruitment of T cells and neutrophils (42, 43). CD137L expression was linked to DC clusters in scRNA-Seq and flow cytometry experiments. Human patients with periodontitis and hyperglycemia had an almost 2-fold increase in CD137L^+^ DCs compared with NG individuals with periodontitis. In vitro data indicated that CD137L was upregulated by exposure to LPS, AGEs, and TNF to much higher levels when simultaneously exposed to HG. It is noteworthy that, to our knowledge, CD137L signaling has not been linked previously to diabetic complications. However, CD137L has been shown to enhance cytotoxic T lymphocyte activation and proliferation in antitumor immunity (26) and may contribute to colitis (44) as well as atherosclerosis in a mouse model (45).

Multiple lines of evidence demonstrate that CD137L produced by DCs is an important factor in the immune dysregulation caused by diabetes. scRNA-Seq analysis showed that the cells with the highest level of CD137L expression were DCs, and the only other cell cluster that expressed CD137L was a macrophage cluster. In diabetic mice, lineage-specific inhibition of Akt1 in DCs reduced CD137L expression in DCs without affecting CD137L expression in other cell types. Furthermore, co-culture in vitro experiments under HG conditions demonstrated that CD137L-blocking Ab inhibits the ability of DCs to activate γδ T cells, demonstrating that CD137L produced by DCs plays an essential role in stimulating γδ T cells (see Figure 9J). Together, these findings suggest DCs are a significant source of CD137L and may play a key role in the local immune dysregulation caused by diabetes. However, the data do not rule out the possibility that other cells, such as macrophages, may also contribute to the pathology caused by dysregulated CD137L production.

scRNA-Seq analysis also indicated that *Cd137,* the cognate receptor for CD137L was highly expressed by γδ T cells and Treg subclusters, which was confirmed by flow cytometry. Notably, diabetes-induced CD137 expression by γδ T cells was reversed by Akt1 deletion in DCs, as shown by scRNA-Seq and flow cytometry. This linkage is further supported by evidence that diabetes increased proliferating γδ T cells in vivo, which was also reversed by DC-specific Akt1 deletion (*P* < 0.05). Lastly, co-culture of DCs with γδ T cells in HG led to an increase in γδ T-cell proliferation that was dependent upon Akt1 expression in DCs. The increased proliferation was blocked by a CD137L antagonist Ab, further establishing a functional relationship.

This evidence suggests diabetes-increased CD137L expression could play a major role in tissue damage increased by diabetes, particularly because it was linked to increased γδ T cells. To investigate this relationship from a functional standpoint, we injected a CD137 agonist that stimulates CD137L receptor signaling. Application of the CD137 agonist to normal mice challenged with bacteria increased periodontal bone loss to the same extent as that observed in diabetic animals. Moreover, it was accompanied by an increase in neutrophils and γδ T cells. We also approached this from a loss-of-function approach in which CD137L was inhibited by a specific blocking Ab in diabetic animals. CD137L blockade rescued diabetes-enhanced bone loss resulting from bacterial challenge, downregulated the number of γδ T cells, and reduced the recruitment of CD45^+^ leukocytes. It also rescued elevated neutrophil recruitment, which is closely tied to greater periodontal tissue loss (46). Collectively, the results indicate the CD137L-CD137 signaling pathway is a driving force in the greater impact of diabetes-enhanced leukocyte recruitment that causes destructive inflammation.

In addition to our in vitro findings demonstrating enhanced DC-mediated activation of γδ T cells under HG conditions, emerging in vivo data further support the critical role of γδ T cells in diabetes-exacerbated periodontal destruction. Previous studies have shown that γδ T cells contribute to pathogen-driven, bone-destructive immune responses in the oral cavity (47). Consistent with this, our recent published data (48) demonstrate that selective inhibition of γδ T cell activation attenuates diabetes-enhanced periodontitis but has no significant effect on periodontal bone loss in NG mice. These results suggest γδ T cells are not only responsive to HG-activated DCs but also act as key mediators of diabetes-specific immune pathology in periodontitis.

To examine how γδ T cells might lead to greater tissue loss, scRNA-Seq results were further analyzed, and pointed to *IL-17a*. *IL-17a* was largely produced by γδ T cells, as shown by positive average log2FC values and a relatively high percentage of cells expressing *IL-17a* in the cluster (pct 1) compared with other clusters (Table 6). The induction of periodontitis considerably enhanced IL-17A expression compared with baseline over a 2- to 6-week period, but the increase in IL-17A expression in diabetic mice was significantly greater than the NG group (Supplemental Table 4). The lineage-specific deletion of Akt1 in DCs reversed the high IL-17A expression in γδ T cells in diabetic periodontitis, as shown by bioinformatic analysis and flow cytometry, suggesting that DCs and Akt1 play a critical regulatory role in controlling IL-17A production in these cells. Furthermore, flow cytometry demonstrated that the CD137 agonist increased IL-17A expression in a manner similar to diabetes, and the increase in IL-17A in the diabetic group was reversed by the CD137L antagonist. This indicates that CD137L signaling modulates IL-17A production, thereby influencing periodontal bone resorption in diabetes. These findings are clinically important because IL-17A is a strong inducer of RANKL expression, which promotes osteoclastogenesis and bone resorption (49).

In addition to these observations, there are other potential mechanisms through which activated γδ T cells or their inhibition could affect periodontal inflammation and bone loss. γδ T cells in gingiva secrete IL-17 and IFN-γ, which can enhance inflammation through stimulating inflammatory macrophage activation in periodontitis (50). Furthermore, γδ T cells can stimulate DCs to produce IL-23, which induces Th17 differentiation and expansion (51, 52). The pro-inflammatory effect on these other cells, in turn, may lead to greater production of RANKL by several cell types, including B cells (50, 53). Because γδ T cells are a major producer of IL-17, a reduction in IL-17 via inhibition of γδ T cell activation may affect inflammation by reducing the pathogenicity of the oral microbiota, as demonstrated when IL-17 is inhibited by Abs in diabetic mice (10). It is also possible that there could be a compensatory increase in IL-17 production by other cell types such as Th17 or ILC3 cells (54). Although we did not observe this effect, it could occur at later points with prolonged inhibition.

A limitation of this study is the use of STZ-induced diabetes, which models type 1 diabetes. Although this model reflects key features of diabetic complications, it does not capture the insulin resistance typical of type 2 diabetes, the more common form of diabetes. However, because both types of diabetes are associated with increased periodontal inflammation and bone loss (9), the findings remain broadly relevant, as supported by in vitro studies that closely tie hyperglycemia, a feature of both type I and type 2 diabetes, to dysregulation of the CD137L-CD137 axis. Furthermore, CD137L expression was elevated in humans with hyperglycemia associated with insulin resistance. Studies using type 2 diabetes models are necessary to investigate the specific linkage to type 2 diabetes.

In conclusion, we identified an exacerbating effect of diabetes on periodontal tissue damage through hyperglycemia-induced dysregulation of DCs and γδ T cells involving the CD137L-CD137 signaling axis. DCs were established as a pivotal factor in this dysregulation because Akt1 ablation specifically in DCs inhibited CD137L expression in vivo and in vitro and significantly reversed the dysregulation in immune-cell populations and bone loss caused by diabetes. The functional role of upregulated CD137L was shown by reversal of enhanced γδ T cell proliferation and recruitment, elevated neutrophil recruitment, and infiltration of cells expressing IL-17A. Interestingly, human gingiva specimens obtained from patients with hyperglycemia had significantly elevated CD137L expression and increased leukocyte recruitment, showing parallels between mice and human tissue samples. Lastly, the results may provide potential insight into the impact of diabetes on other inflammation-driven pathologies.

## Methods

### Sex as a biological variant.

Male and female mice were used in equal numbers across all experimental groups. For flow cytometry and in vitro assays, gingival tissues from 1 male and 1 female mouse were pooled to generate each biological replicate. For microCT and histologic analyses, male and female mice were evaluated separately, with no trends of sex-based difference observed in any measured outcomes. Similarly, no sex-based trends were noted in studies with human gingival tissue.

### Study design.

We tested whether hyperglycemia dysregulates host responses to bacterial challenge and increases inflammatory damage. Periodontitis was induced by oral inoculation with *P*. *gingivalis* and *F*. *nucleatum*. scRNA-Seq profiled leukocytes and highlighted DCs, γδ T cells, and CD137L–CD137 signaling. Akt1 function in DCs was tested with lineage-specific deletion (CD11c.Cre^+/−^.AKT1^LL^) versus controls (CD11c.Cre^−/−^.AKT1^LL^). Endpoints were periodontal bone loss and immune responses under NG or Diab conditions. Diabetes was induced with low-dose STZ. Findings were validated by flow cytometry or immunofluorescence in mice and human gingiva. microCT and histology were used to quantify periodontal damage. CD137L–CD137 signaling as a mechanistic basis for diabetes-enhanced damage was tested in vivo with agonist or antagonist Abs. Each data point is from an independent experiment.

### CD11c.Cre.AKT1^L/L^ experimental mouse models.

CD11c.Cre^+^ mice were crossed with AKT1^LL^ mice to generate CD11c.Cre^+/−^.AKT1^LL^ (experimental) and CD11c.Cre^−/−^.AKT1^LL^ (control) littermates (55). Cre recombinase mice under control of the CD11c promoter were purchased from The Jackson Laboratories. AKT1^LL^ mice were provided by Morris Birnbaum (Perelman School of Medicine, University of Pennsylvania, Philadelphia PA). Genotypes were confirmed by PCR. Mice were housed in specific pathogen-free conditions with ad libitum chow and water.

### Diabetes induction and experimental groups.

STZ (40 mg/kg) was given intraperitoneally for 5 consecutive days; control mice received citrate buffer (56, 57). Mice were considered diabetic when their blood glucose concentration was >220 mg/dL; studies began after ≥4 weeks of hyperglycemia. There were 3 groups (*n* = 6/group): NG CD11c.Cre^−/−^.AKT1^LL^ mice, diabetic CD11c.Cre^−/−^.AKT1^LL^ mice, and diabetic CD11c.Cre^+/−^.AKT1^LL^ mice.

### Periodontitis induction.

Bacteria *P*. *gingivalis* (ATCC, 33277) and *F*. *nucleatum* (ATCC, 25586) were cultured anaerobically at 37°C and prepared for inoculation. Mice received antibiotics in water for 8 days and then had a 2-day washout. Inoculations with *P*. *gingivalis* and *F*. *nucleatum* (10^9^ CFU each in 100 μL 2% carboxymethyl cellulose/PBS) were given 3 times weekly for 2 weeks.

### CD137L gain- and loss-of-function mouse models.

NG mice received a weekly intraperitoneal isotype control or a CD137 agonist starting on day 1 of inoculation (*n* = 6 inoculations total). Diab WT mice received an isotype control or a CD137L antagonist, initiated 3 days before inoculation and then weekly (*n* = 7 total). There were 6 mice per group. Mice were euthanized 4 weeks after the last inoculation. Gingiva specimens were collected for flow cytometry, maxillae for microCT, and mandibles for histology and immunofluorescence.

### Human specimen collection.

Gingival tissues were obtained from patients undergoing periodontal surgery. Patients had periodontitis stage II/III with bone loss and probing depths ≥5 mm. Inclusion criteria were age 30–70 years, nonsmokers, and having ≥10 teeth. Individuals with hyperglycemia (*n* = 4) had HbA_1c_ >6.75%; NG individuals (*n* = 4) had HbA_1c_ ≤5.5%. Exclusion criteria were pregnancy or breastfeeding, inflammatory or autoimmune disease, immunosuppression, smoking >5/day, recent antibiotics, or being nonambulatory.

### Mouse gingival tissue processing for single-cell isolation and scRNA-Seq.

Gingiva specimens (~1.5 mm around all molars) from 4 mice per group were pooled, minced, and digested with collagenase, dispase, and DNase (37°C, 45 minutes). Cells were filtered, stained, and sorted to enrich CD45^+^CD11c^−^, CD45^+^CD11c^low^, and CD45^+^CD11c^high^ fractions (8:1:1). 10,000 cells/group were then loaded for barcoded cDNA libraries on the Chromium Single Cell 3′ platform (version 3; 10X Genomics. Quality metrics are in Supplemental Table 1.

### Histologic analysis.

Mandibular molars and periodontium were fixed, decalcified in 10% EDTA at 4°C for approximately 4 weeks, embedded in paraffin, sectioned at 5 μm, and mounted. Sections were stained with H&E or for immunofluorescence (58). Connective tissue attachment loss was from the CEJ to epithelial attachment; alveolar bone loss was from the CEJ to the alveolar crest (59, 60). For IL-17A immunofluorescence, paraffin sections were treated with heat-induced antigen retrieval at 120°C in Tris-EDTA buffer (pH 9), and a standard immunostaining protocol was followed using a specific primary antibody and a secondary antibody conjugated with a fluorescent signal. IL-17A^+^ cells/mm^2^ were quantified by image analysis.

### Flow cytometry analysis of mouse gingival tissue.

Gingiva were excised, washed, minced, and enzymatically digested as described above. Cell suspensions were filtered, Fc-blocked, and stained (Supplemental Table 2) with live/dead cell exclusion. Samples acquired by flow cytometry gating excluded debris (forward scatter [FSC]/side scatter [SSC]), doublets (FSC area vs. FSC height), and dead cells; viable leukocytes were Zombie^−^ CD45^+^. FMO controls defined gating strategies which are shown in Supplemental Figures 1–6.

### microCT scanning, image reconstruction, and analysis.

Fixed maxillae were scanned by microCT (voxel, ~9 μm; 55 keV; 300 ms). Reconstructions were analyzed to measure the distance from the CEJ to the alveolar bone crest and percent bone in the interdental area between the first and second maxillary molars. For every mouse, values were averaged from 3 slices from the buccal, palatal, and middle regions, and from both sides.

### Human tissue flow cytometry.

Fresh gingival tissues were processed (i.e., minced, digested [37°C, 1 hour], filtered, RBC lysed, and Fc blocked) within 2 hours of surgical incision. Viability dye was used to distinguish live cells from dead cells. Cells were stained with CD45, HLA-DR, CD11c, and CD137L antibodies. Flow cytometry data were acquired on LSRII and analyzed on FlowJo by using FMO controls to guide gating strategy as above.

### Isolation and stimulation of BMDCs.

BMDCs were generated from femurs of 4- to 6-week-old C57BL/6 mice or from CD11c.Cre^+/−^.AKT1^LL^ and Cre^–^littermates, and cultured in RPMI with 10% FBS and GM-CSF. After differentiation, BMDCs were maintained for 5 days in NG (5 mmol/L) or HG (25 mmol/L) conditions and, where indicated, stimulated with LPS (24 hours), AGE-BSA (5 days), or TNF (5 days).

### Isolation and culture of γδ T cells.

Spleens from 6- to 10-week-old C57BL/6 mice were processed to single cell suspension, red blood cells were lysed, and γδ T cells were purified by magnetic-activated cell sorting (Miltenyi). Cells were cultured in RPMI with 10% FBS and IL-2, activated on plate-bound anti-CD3 and anti-CD28, and then expanded for 5 days.

### Co-culture of DCs and γδ T cells.

BMDCs and γδ T cells were co-cultured at a 1:4 ratio in NG or HG medium. Cultures received agonistic anti-CD137 or antagonistic anti-CD137L (20 ng/mL) for 5 days, then were stimulated with PMA, ionomycin, and brefeldin A for 6 hours before flow cytometry. Antibody panels are listed in Supplemental Table 2.

### qPCR analysis.

RNA was isolated and reverse transcribed to prepare a cDNA library. RT-qPCR quantified IL-17a, Tnfsf9, Ccl3, and Ccl4, normalized to L32, by the comparative Ct method.

### Flow cytometry analysis for in vitro studies.

DCs and γδ T cells were Fc blocked and stained with viability dye. Surface staining included CD11c and TCRγδ. For intracellular IL-17A and Ki-67, cells were fixed, permeabilized, and stained for flow cytometry data acquisition and analysis as described above. Live TCRγδ^+^ cells were pregated for IL-17A and Ki-67 (Supplemental Figure 5).

### Statistics.

Data were analyzed using 1-way ANOVA and Tukey’s post hoc test to determine the interaction effects between different factors on the measured outcomes. The significance level was set at *P* < 0.05. All statistical analyses were performed using GraphPad Prism, version 9.3.1. Data are presented as mean ± SD, and statistical significance is denoted where applicable. All *P* values were corrected for multiple comparisons, using the Benjamini-Hochberg method.

### Study approval.

All animal procedures were conducted in accordance with ethical guidelines and approved by the University of Pennsylvania IACUC to ensure the humane treatment of animals and compliance with relevant regulations. Human gingival tissues were collected from consenting patients undergoing periodontal surgery at the periodontal clinics, School of Dental Medicine, University of Pennsylvania. The study received IRB approval (IRB 843434, University of Pennsylvania). Written informed consent was obtained from all participants prior to sample collection, in accordance with the Declaration of Helsinki.

### Data availability.

All sequencing data generated in this study have been deposited in the Gene Expression Omnibus under accession number GSE295144 and are publicly available. Supporting data values underlying the figures and results are provided in the Supporting Data Values accompanying this article. Analytic scripts and additional datasets used and/or analyzed during this study are available from the corresponding author upon reasonable request.

## Author contributions

XH and ML performed the experiments, analyzed data, and drafted the manuscript. MVG conducted bioinformatic analyses. RD performed the in vitro experiments. HA performed the IF experiments. SAK performed H&E staining and histological scoring. YC revised the manuscript and figures. DTG conceived the project, designed and supervised the experiments, analyzed the data, and revised the manuscript. KIK designed and supervised the experiments, interpreted the data, and revised the manuscript.

## Funding support

This work is the result of NIH funding, in whole or in part, and is subject to the NIH Public Access Policy. Through acceptance of this federal funding, the NIH has been given a right to make the work publicly available in PubMed Central.

- US National Institute of Dental and Craniofacial Research (grants R01DE021921 and R01DE017732 to DTG, and R01DE030415 to KIK).
- The National Science Foundation of China (grant 82201056 to XH).

## Supplementary Material

Supplemental data

Supporting data values

## Figures and Tables

**Table 3:**
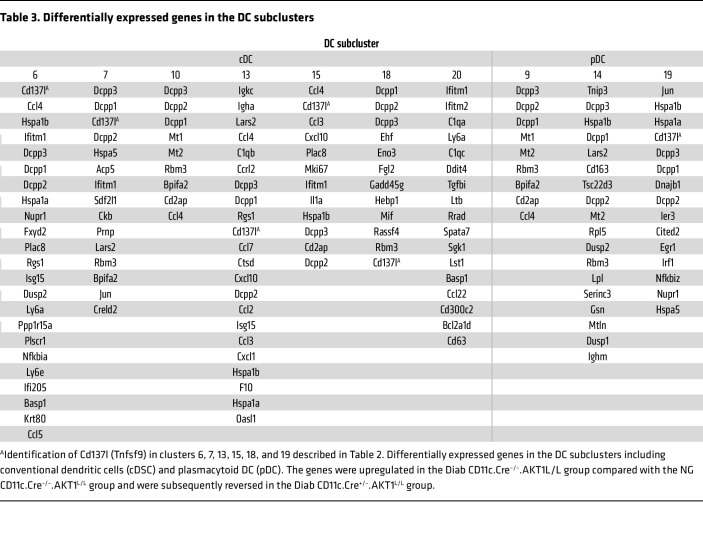
Differentially expressed genes in the DC subclusters

**Table 4:**
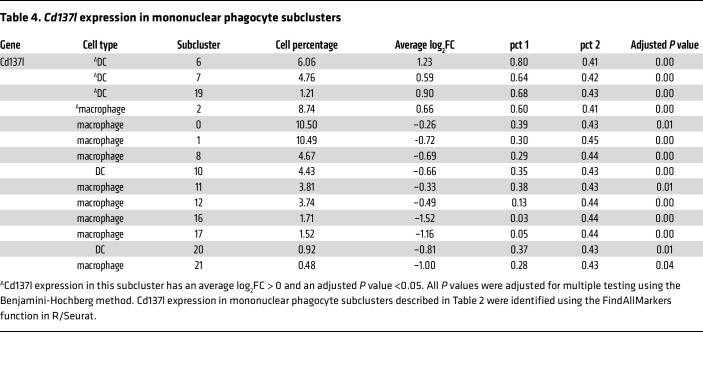
*Cd137l* expression in mononuclear phagocyte subclusters

**Table 5:**
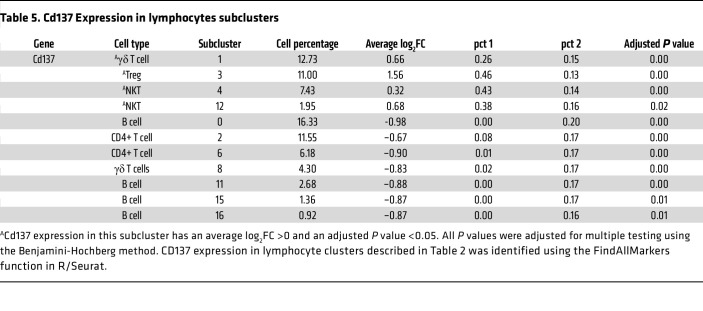
Cd137 Expression in lymphocytes subclusters

**Table 7:**
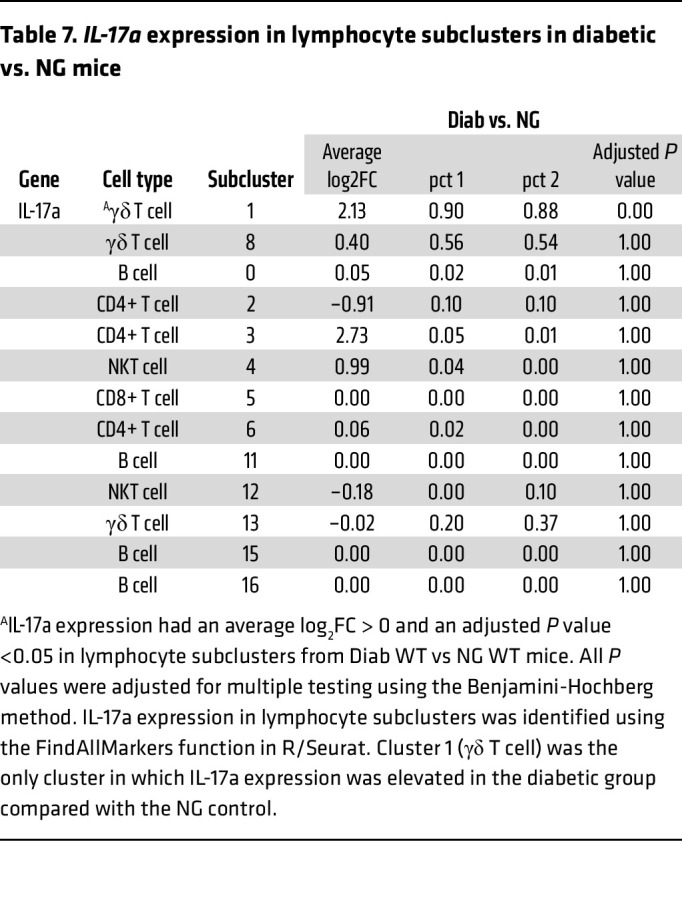
*IL-17a* expression in lymphocyte subclusters in diabetic vs. NG mice

**Table 6:**
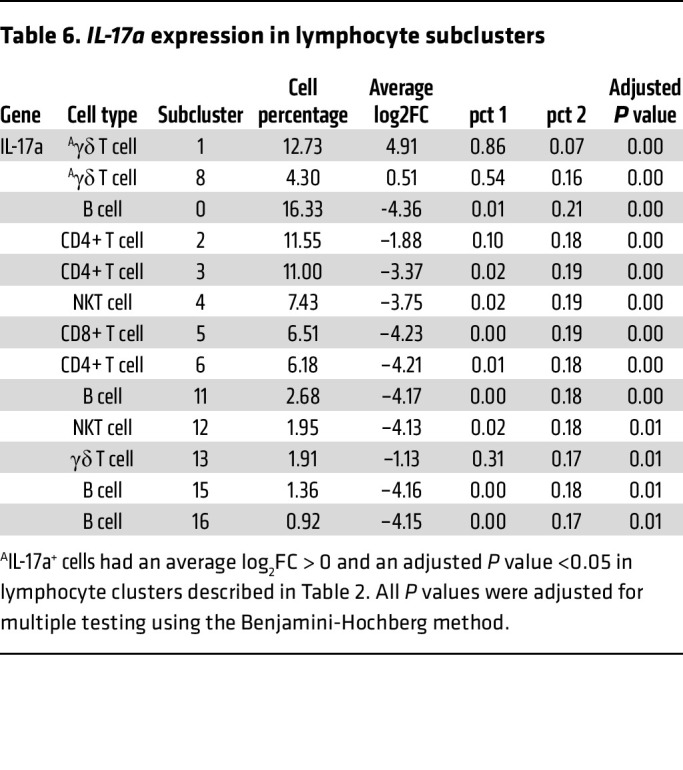
*IL-17a* expression in lymphocyte subclusters

**Table 2:**
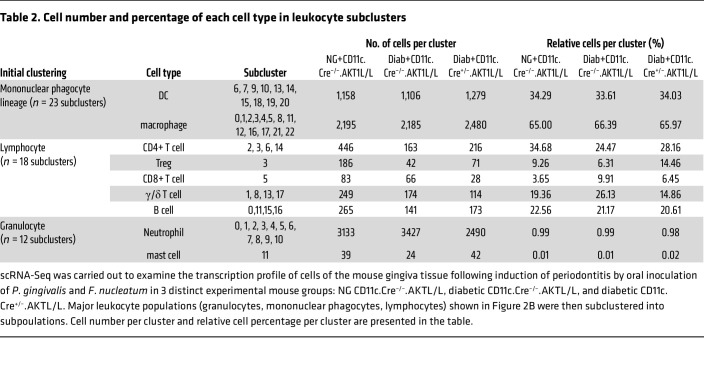
Cell number and percentage of each cell type in leukocyte subclusters

**Table 1:**
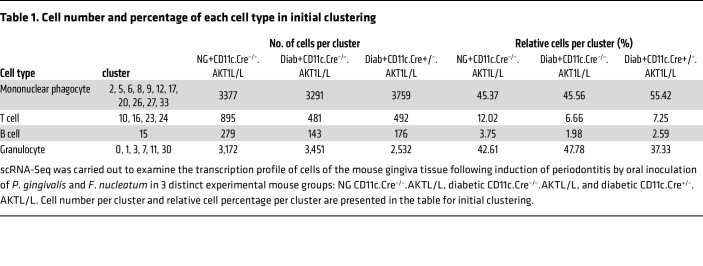
Cell number and percentage of each cell type in initial clustering

## Version 1 12/11/2025

In-Press Preview

## Version 2 02/02/2026

Electronic publication
