# Supplementary material for: Diabetes exacerbates destructive inflammation by activating the CD137L-CD137 axis in dendritic and IL-17+ T cells
Source: J Clin Invest. 2025 Dec 11;136(3):e193289. doi: 10.1172/JCI193289 (PMC12867133; doi:10.1172/JCI193289)
Supplement: Supplemental data [file jci-136-193289-s249.pdf]

Supplemental Methods

Study Design

This study investigates the role of hyperglycemia in causing dysregulation of the host response leading to increased inflammatory tissue damage induced by bacterial challenge. Periodontitis was induced by oral inoculation of the pathogens *P gingivalis* and *F nucleatum*. We utilized scRNAseq to identify leukocyte alterations at the cellular and molecular levels, which led us to focus on dendritic cells, γδ T-cells and CD137L-CD137 interactions. The role of Akt1 in modulating dendritic cell function was examined through mice with lineage-specific Akt1 deletion in experimental CD11c.Cre$^{+/-}$.Akt1$^{L/L}$ or control CD11c.Cre$^{-/-}$.Akt1$^{L/L}$ mice. We conducted in vivo experiments to assess periodontal bone loss and immune responses under normoglycemic and diabetic conditions. Diabetes was induced in mice with multiple low-dose streptozotocin injection. scRNAseq results were validated by flow cytometry or immunofluoresence in murine or human specimens obtained from subjects with hyperglycemia associated with type-2 diabetes. MicroCT and histologic analysis were used to assess tissue damage. To further dissect the functional role of CD137L-CD137 signaling, a specific agonist and specific antagonist were used to evaluate their effects on inflammation and bone loss. Each data point in the bar graphs represents a separate independent experiment.

CD11c.Cre.AKT$^{L/L}$ Experimental mice models.

CD11c.Cre mice were purchased from the Jackson Laboratory (Stock # 8068; B6; Bar Habor, ME, USA), which express Cre recombinase under control of the integrin alpha X gene (*Itgax*) promoter/enhancer region. AKT1$^{L/L}$ mice were a generous gift from Dr. Morris Birnbaum and have been described in Ref (1). AKT1$^{L/L}$ mice were bred with CD11c.Cre mice to generate experimental

mice ($CD11c.Cre^{+/-}.AKT1^{L/L}$) and the control mice littermates ($CD11c.Cre^{-/-}.AKT1^{L/L}$). Genotyping was performed using polymerase chain reaction (PCR) analysis of tail DNA samples to confirm the presence of the Cre and $AKT1^{L/L}$ alleles. Mice were housed in specific pathogen-free conditions with controlled temperature, humidity, and a 12-hour light-dark cycle. Standard rodent chow and water were provided ad libitum. All animal procedures were conducted in accordance with ethical guidelines and approved by the University of Pennsylvania Institutional Animal Care and Use Committee to ensure the humane treatment of animals and compliance with relevant regulations.

Diabetes was induced by multiple low dose intraperitoneal (i.p.) injections (2) of streptozotocin (40 mg/kg; Sigma, St Louis, MO, USA) in 10 mmol sodium citrate buffer (pH=4.5) for 5 days as we have previously described (3). Control mice were treated identically with an equal volume of citrate buffer. Mice were considered to be hyperglycemic when blood glucose levels were >220 mg/dl and experiments initiated when mice were hyperglycemic for at least 4 weeks. Three groups were examined: normoglycemic $CD11c.Cre^{-/-}.AKT^{L/L}$, diabetic $CD11c.Cre^{-/-}.AKT^{L/L}$, and diabetic $CD11c.Cre^{+/-}.AKT^{L/L}$. Each group consisted of 6 mice, with age and sex matched across groups.

Periodontitis was induced by oral inoculation with *Porphyromonas gingivalis* (ATCC #33277) and *Fusobacterium nucleatum* (ATCC #25586). Mice were initially given antibiotic in water ad libitum (sulfamethoxazole 0.87 mg/ml and Trimethoprim 0.17 mg/ml) for 8 days followed by 2 days without antibiotics and then subjected to oral inoculation with *P gingivalis* and *F nucleatum* (10^9 colony-forming units of each bacterium in 100 μL of 2% carboxymethyl cellulose in PBS) directly into the oral cavity three times per week for two weeks.

CD137L Gain and Loss of Function Mouse Animal Model.

Normoglycemic CD11c.Cre$^{-/-}$.AKT$^{L/L}$ received i.p. injections (2, 4, 5) of 200 µg of rat IgG2a κ isotype control antibody (Biolegend #400566) or agonist antibody specific to CD137 (rat MAb, BioXcell #BP0239) are described in ref (6, 7) that mimics CD137L activation of CD137. The injection was started at the day of oral inoculation with *P gingivalis* and *F nucleatum* as described above and subsequently once per week until euthanize, 6 times total. Diabetic CD11c.Cre$^{-/-}$.AKT$^{L/L}$ received i.p. injections of 200 µg rat IgG2a, κ isotype control antibody (Biolegend #400566) or 300 µg of CD137L antagonist antibody that blocks activation of CD137 (Biolegend #107114) (8). The injection was initiated 3 days before oral inoculation and subsequently once per week until euthanasia for a total of 7 injections. All the antibodies were diluted in sterilized PBS.

Each group consisted of 6 mice, with age and sex matched across groups. The animals were euthanized 4 weeks after the day of oral inoculation was completed. The gingiva from the maxilla was harvested and cells isolated as described above for flow cytometry. The remaining bone of the maxilla was fixed in 4% paraformaldehyde overnight and bone levels were examined by microCT. The molars and associated periodontal tissue in the mandibles were processed for histologic and immunofluorescent analysis.

Human specimen collection

Gingival tissues were collected from consenting patients undergoing periodontal surgery at the periodontal clinics, School of Dental Medicine, University of Pennsylvania. The study was approved by Institutional Review Board approval (IRB #843434). Participating patients were diagnosed with periodontitis stage II/III and had radiographic evidence of bone loss and periodontal pocket depths ≥5 mm. Inclusion criteria were non-smokers, age 30-70 years, in good

general health, a minimum of 10 teeth, and willingness to follow study procedures. Individuals with hyperglycemia (N=4) had HbA1c >6.75% linked to insulin resistance, while normoglycemic subjects (N=4) had HbA1c ≤5.5%. Exclusion criteria included age <30 years, pregnancy or breastfeeding, chronic inflammatory or autoimmune diseases, immunosuppressive medication use, non-ambulatory status, smoking >5 cigarettes/day, and antibiotic treatment within three months of tissue collection.

Bacteria

Periodontitis was induced by oral inoculation with *Porphyromonas gingivalis* (*Pg*, #33277; ATCC) and *Fusobacterium nucleatum* (*Fn*, #25586; ATCC). Both bacteria were cultured separately in Brain Heart Infusion (BHI) (BD BACTO™ Cat#237500) broth and maintained in anaerobic conditions using an anaerobic jar at 37°C. The cultures were passed daily. Anaerobic conditions were generated using Anaerogen (BD Gaspak™ EZ anaerobe container system sachets with indicator, Cat#260001).

Mouse Gingival Tissue processing for single-cell isolation and scRNAseq analysis.

Single-cell RNA sequencing (scRNA-seq) was carried out to examine the transcription profile of cells of the mouse gingiva tissue following induction of periodontitis by oral inoculation of *P gingivalis* and *F nucleatum* in three distinct experimental groups: normoglycemic CD11c.Cre$^{-/-}$.AKT$^{L/L}$, diabetic CD11c.Cre$^{-/-}$.AKT$^{L/L}$, and diabetic CD11c.Cre$^{+/-}$.AKT$^{L/L}$. The gingival tissue (~1.5mm) surrounding all of the molars was carefully dissected under the microscope. The gingiva obtained from 4 mice for each experimental group were pooled, minced and subjected to 3.2 mg/mL Collagenase IV (Worthington), 2.65 mg/mL Dispase II (Sigma-Aldrich) and 0.15 mg/mL

DNase I (Sigma-Aldrich) at 37°C for 45 minutes and strained to remove large debris. Cells were incubated with DAPI (Abcam Ca#ab104139) and antibodies specific for CD45 and CD11c and sorted to remove DAPI$^+$ dead cells, and to obtain cell populations that were enriched in CD45$^+$CD11c$^-$ cells, CD45$^+$CD11c^{low} cells and CD45$^+$CD11c^{high} cells in an 8:1:1 ratio. Approximately 10,000 cells were loaded per lane for each group and barcoded cDNA libraries using the 10X Genomics Chromium Single Cell 3' Solution (v3) were created according to the manufacturers protocol. The quality control assessment of scRNA-seq transcripts were excellent as shown in Table S1.

Histologic Analysis

The mandibular molar teeth and associated periodontal tissue was obtained, fixed in 10% neutral-buffered formalin for 24 hours, and subsequently decalcified in 10% ethylenediaminetetraacetic acid (EDTA) at at 4°C in a cold room on a shaker for 4 weeks, with the decalcifying solution changed every other day. Following decalcification, the tissues were sequentially dehydrated through a graded series of ethanol (70%, 80%, 95%, and 100%), cleared in xylene, and embedded in paraffin wax. Paraffin blocks were sectioned at a thickness of 5 µm using a microtome, and the sections were mounted onto positively charged glass slides. The sections were examined by histostains including hematoxylin and eosin (H&E) or prepared for immunofluorescence as described below.

For H&E staining, sections were deparaffinized in xylene (2 x 10 min) and rehydrated through a graded ethanol series (100%, 95%, 80%, 70%) to distilled water. Slides were stained with hematoxylin and then eosin as described in ref (9). The cementoenamel junction was used as a fixed reference point since it is readily visible in H & E stained decalcified sections. Loss of

connective tissue attachment was measured in H & E stained sections as described in using the distance from the cementoenamel junction to the height of the epithelial attachment as in ref (10, 11). Immunofluorescence was carried out to examine the expression of IL-17A in paraffin-embedded 5 µm sections of mouse mandibles with gingiva. Antigen retrieval was performed by heating the sections in Tris-EDTA (pH 9) at 95-100°C for 10 minutes, followed by and sections were then incubated with blocking agent (5 % donkey serum in 1×PBS,) for 1 hour, at room temperature. The sections were incubated with antibody against interleukin (IL)-17A (concentration: 4 µg/mL, dilution: 1:112.5, Proteintech Group, Cat#26163-1-AP) or matched control antibody (Rabbit IgG, concentration: 4 µg/mL, dilution: 1:1250, Vector Labs, Cat#I-1000-5) for 2 hours at room temperature in a humidified chamber and then localized with donkey anti-rabbit IgG (concentration:3 µg/mL, 1:500 dilution; Jackson ImmunoResearch Cat#711-605-152) conjugated with Alexa Fluor® 647. Sections were then mounted with Fluoroshield Mounting Medium with DAPI (Abcam, Cat#ab104139) and covered with glass coverslips. Image analysis was performed using NIS Elements [AR 5.20.02 64-bit] software to assess IL-17A positive cells per area (mm2) in the mouse gingival tissues.

Flow Cytometry Analysis of Mouse Gingival Tissue.

Gingival tissues were harvested from a murine model under sterile conditions. Briefly, mice were euthanized, and the maxilla were dissected to expose the gingiva. Gingival tissues were carefully excised and washed in phosphate-buffered saline (PBS) to remove blood and debris. To obtain single-cell suspensions, gingiva tissues were minced into small fragments and enzymatically digested using a cocktail of DNAseI (Sigma-Aldrich, Cat#10104159001), Collagenase IV (Worthington, Cat#LS004188) and Dispase II (Sigma-Aldrich, Cat#D4693-1G). After digestion,

cells were filtered through a 70 µm cell strainer. Single-cell suspensions from gingival tissues were incubated with Fc blocker (Biolegend Cat# 101302) to block the non-specific binding. Cells were then incubated with fluorochrome-conjugated antibodies (refer to ab list in Table S2) against surface markers in PBS with 2.5% FBS, for 30 minutes at 4°C in the dark, and then washed. Dead cells were excluded with Live/Dead fixable dye (Zombie yellow, 1:100, Biolegend Cat #423104). Antibodies were titrated to determine optimal staining concentrations. Cell acquisition was performed in LSRII (BD Biosciences, San Jose, CA) and analyzed using Flowjo (v10). Gating strategies were established based on fluorescence minus one (FMO) controls and isotype controls. In detail, initial gating was performed to exclude debris and to identify the main cell population based on cell size and granularity. Cells with low FSC and SSC values were gated out. The single cell population was identified by plotting FSC-A (area) vs. FSC-H (height) to exclude doublets. Only single T-cells were included in subsequent analysis. Live and dead leukocytes were distinguished using a Zombie viability dye (Biolegend). The viable leukocytes population was identified as Zombie$^-$CD45$^+$. Fluorescence Minus One (FMO) Controls were utilized for each antibody. Data were analyzed to quantify immune cell populations and characterize their activation status within the gingival tissue. (detailed gating strategies and FMOs for each flow cytometry were shown in Figure S1-S6)

MicroCT scanning, image reconstruction and analysis.

To examine alveolar bone loss, microcomputed tomography (micro-CT) analysis was performed on maxillary bones obtained from a murine model after *P gingivalis/F nucleatum* inoculation. The maxillae were carefully dissected and fixed in 10% neutral buffered formalin for 24 hours at 4°C to preserve their structure. Fixed maxillary bones were scanned using Scanco

Medical μCT 45 at the Imaging Core, Penn Center for Musculoskeletal Disorders. Scans were performed at 55 keV (9 μm, 300 ms) to ensure optimal image quality. The 3D images were then reconstructed and analyzed using OsiriX MD software (Version 12.0, Pixmeo, Geneva, Switzerland). Bone loss was measured by two different radiographic approaches. One was the distance from the consistent landmark on the tooth surface, the cementoenamel junction (CEJ) to the height of the alveolar bone referred to as the alveolar bone crest. In the second, bone was also quantified as the percent bone in the interdental space between first and second maxillary molar teeth divided by the total area of this space (12). The results of each sample were averaged from three representative slices from buccal, palatal, and middle regions, as well as from the left and right sides of the maxilla for each mouse.

Figure 1A presents a schematic overview of the experimental workflow, detailing the construction of mouse models, micro-CT analysis of the maxilla, followed by cell sorting and single-cell RNA sequencing using the 10X Genomics platform.

Human Tissue flow cytometry

Human gingival tissues were processed within 2 hours of collection for flow cytometry analysis. Briefly, tissues were minced and digested at 37°C with constant agitation in RPMI containing DNAse I (0.15 mg/ml; Roche), collagenase type IV (3.2 mg/ml; Worthington), and dispase (2.6 mg/ml; Sigma-Aldrich) for one hour. Clumps and debris were removed by filtering cells through a 70-μm mesh, and red blood cells were lysed with Ammonium-Chloride-Potassium buffer. Fc receptors were blocked with CD16/32 antibody, and viability dye (Zombie Yellow) was used to distinguish live and dead cells. Cells were stained with a cocktail of antibodies (CD45, HLA-DR,

CD11c and CD137L). Data were acquired using BD LSR II Flow Cytometer and analyzed using FlowJo software. Fluorescence-minus-one controls were used to determine the gating strategy.

Isolation and stimulation of bone marrow-derived dendritic cells.

Primary bone marrow-derived DCs (BMDCs) were generated from bone marrow collected from the femurs of 4-6 weeks old C57BL/6 mice as described previously(13). In another set of experiments, CD11c.Cre$^{+/-}$.AKT$^{L/L}$ mice and control littermates that lack Cre transgene were used. Cells were seeded at a density of 1.5×10^{6} cells in 6-well plates using RPMI-1640 medium supplemented with 1% antibiotics/antimycotics (Gibco, cat#15240-62), 10% heat-inactivated FBS (Gibco, cat#16140071) containing 20 ng/ml GM-CSF (R&D systems, cat#415-ML-005/CF). After differentiation, BMDCs were incubated in either NG condition (5 mmol/L, d-glucose, Sigma, cat#G7021) or high glucose condition (25 mmol/L) for 5 days. In some groups, BMDCs were stimulated with varying concentrations of lipopolysaccharide (Sigma-Aldrich, cat#: L4391) for 24hr, an advanced glycation end-product (AGE-BSA, Cayman chemicals, cat#22968) for 5 days, or tumor necrosis factor-α (TNF-α, R&D systems, cat#410-MT-010/CF) for 5 days prior to experimental endpoint.

Isolation and culture of γδ T-cell.

Spleen was collected from 6-10 weeks old C57BL/6 mice, minced, strained (VWR, cat#76327-100), and RBC was lysed using Ammonium-Chloride-Potassium (ACK) buffer (Quality Biologicals, cat#118156101). γδ T-cells were purified by positive selection with magnetic beads per manufacturer's protocol (Cat# 130-092-125, Miltenyi Biotec, Germany). Cells were

resuspended into RPMI 1640 containing 10% FBS, recombinant mouse IL-2 (5ng/ml) (R&D systems, cat# 402-ML-020/CF) and activated using plate-bound anti-CD3 (1ug/ml) (Invivo, cat#BE0002) and anti-CD28 (5ug/ml) (Invivo, cat#BE0015-5). Cells were expanded for an additional 5 days and used for in vitro assays.

Co-culture of dendritic cells and γδ T-cell.

BMDCs and γδ T-cells were co-cultured at a ratio of 1:4 (BMDC: γδ T-cell) in the RPMI 1640 media that had either high glucose or NG concentrations. Co-cultured cells were treated with agonistic CD137 antibody (BioXcell, #BP0239), or antagonistic CD137L antibody (Biolegend #107114) at 20ng/mL for 5 days followed by incubation with stimulation cocktail that contains PMA (50ng/mL), ionomycin (760ng/mL) and brefeldin A (5µg/mL, Biolegend; #423303) for 6 hours. Cells were then centrifuged and stained with fluorescence-tagged antibodies for flow cytometry analysis. The list of antibodies is available in supplemental Table S2.

Q-PCR analysis

Total RNA was extracted using the RNAqueous Total RNA Isolation Kit (Thermo Fisher scientific, cat#AM1912) and cDNA library preparation with high-capacity cDNA synthesis kit (Applied Biosystems, cat#4368814) as per manufacturer's protocols. Real-time quantitative PCR (RT-qPCR) was performed using Fast Power SYBR Green (Applied Biosystems, cat#4385612) on a Step-One Plus machine (Thermo Fisher Scientific) and normalized by L32 housekeeping gene expression. The primer sets used were as follows: IL-17α (forward 5-GGC CCT CAG ACT ACC TCA AC-3; reverse 5-TCT CGA CCC TGA AAG TGA AGG-3),TNFSF9 (forward 5-CGG CGC

TCC TCA-GAG-ATA-C-3; reverse 5-ATC-CCG AAC ATT AAC CGC AGG-3), Ccl3 (forward 5-TTC TCT GTA CCA TGA CAC TCT GC-3; reverse 5-CGT GGA ATC TTC CGC CGG CTG TAG-3), Ccl4 (forward 5-TTC CTG CTG TTT CTC TTA CAC CT-3; reverse 5-CTG TCT GCC TCT TTT GGT CAG-3), L32 (forward 5-GCC CAA GAT CGT CAA AAA GAG A-3; reverse 5-TCC GCC AGT TAC GCT TAA TTT-3). Relative gene expression was calculated using the delta-delta comparative threshold cycle algorithm ($2^{-\Delta\Delta CT}$) method.

Flow Cytometry Analysis for in vitro study

DCs and γδ T-cells were harvested as described above. Prior to staining, Fc receptors were blocked with CD16/32 antibody, and viability dye (Zombie Yellow™, Biolegend) was used to distinguish live and dead cells. CD11c and TCRγδ antibodies were used for surface antigen staining following standard protocols. For intracellular detection of IL17A and Ki67, cells were fixed at room temperature for 15 min followed by intracellular staining with antibodies in permeabilization buffer for 30 mins. Supplemental Table 2 lists antibody details. Flow cytometry data was acquired as described, and live TCRγδ cells were pre-gated for IL17A and Ki67 expression analyses. (Figure S7)

References

1. Pauta M, Rotllan N, Fernandez-Hernando A, Langhi C, Ribera J, Lu M, et al. Akt-mediated foxo1 inhibition is required for liver regeneration. *Hepatology.* 2016;63(5):1660-74.
2. Al Shoyaib A, Archie SR, and Karamyan VT. Intraperitoneal Route of Drug Administration: Should it Be Used in Experimental Animal Studies? *Pharm Res.* 2019;37(1):12.
3. Furman BL. Streptozotocin-Induced Diabetic Models in Mice and Rats. *Curr Protoc Pharmacol.* 2015;70:5 47 1-5 20.

4. Mikecs B, Vag J, Gerber G, Molnar B, Feigl G, and Shahbazi A. Revisiting the vascularity of the keratinized gingiva in the maxillary esthetic zone. *BMC Oral Health.* 2021;21(1):160.
5. Gupta S, Zhou F, Greer CE, Legg H, Tang T, Luciw P, et al. Antibody responses against HIV in rhesus macaques following combinations of mucosal and systemic immunizations with chimeric alphavirus-based replicon particles. *AIDS Res Hum Retroviruses.* 2006;22(10):993-7.
6. Qi X, Li F, Wu Y, Cheng C, Han P, Wang J, and Yang X. Optimization of 4-1BB antibody for cancer immunotherapy by balancing agonistic strength with FcgammaR affinity. *Nat Commun.* 2019;10(1):2141.
7. Shuford WW, Klussman K, Tritchler DD, Loo DT, Chalupny J, Siadak AW, et al. 4-1BB costimulatory signals preferentially induce CD8+ T cell proliferation and lead to the amplification in vivo of cytotoxic T cell responses. *J Exp Med.* 1997;186(1):47-55.
8. Akiba H, Miyahira Y, Atsuta M, Takeda K, Nohara C, Futagawa T, et al. Critical contribution of OX40 ligand to T helper cell type 2 differentiation in experimental leishmaniasis. *J Exp Med.* 2000;191(2):375-80.
9. Hiyari S, Wong RL, Yaghsezian A, Naghibi A, Tetradis S, Camargo PM, and Pirih FQ. Ligature-induced peri-implantitis and periodontitis in mice. *J Clin Periodontol.* 2018;45(1):89-99.
10. Kimura F, Miyazawa K, Hamamura K, Tabuchi M, Sato T, Asano Y, et al. Suppression of alveolar bone resorption by salubrinal in a mouse model of periodontal disease. *Life Sci.* 2021;284:119938.
11. Gao R, Zhang W, Jiang Y, Zhai J, Yu J, Liu H, and Li M. Eldecalcitol effectively prevents alveolar bone loss by partially improving Th17/Treg cell balance in diabetes-associated periodontitis. *Front Bioeng Biotechnol.* 2023;11:1070117.
12. Xiao E, Mattos M, Vieira GHA, Chen S, Correa JD, Wu Y, et al. Diabetes Enhances IL-17 Expression and Alters the Oral Microbiome to Increase Its Pathogenicity. *Cell Host Microbe.* 2017;22(1):120-8 e4.
13. Sauter M, Sauter RJ, Nording H, Olbrich M, Emschermann F, and Langer HF. Protocol to isolate and analyze mouse bone marrow derived dendritic cells (BMDC). *STAR Protoc.* 2022;3(3):101664.

Figure S1: Histologic and microCT analyses of periodontal tissues.

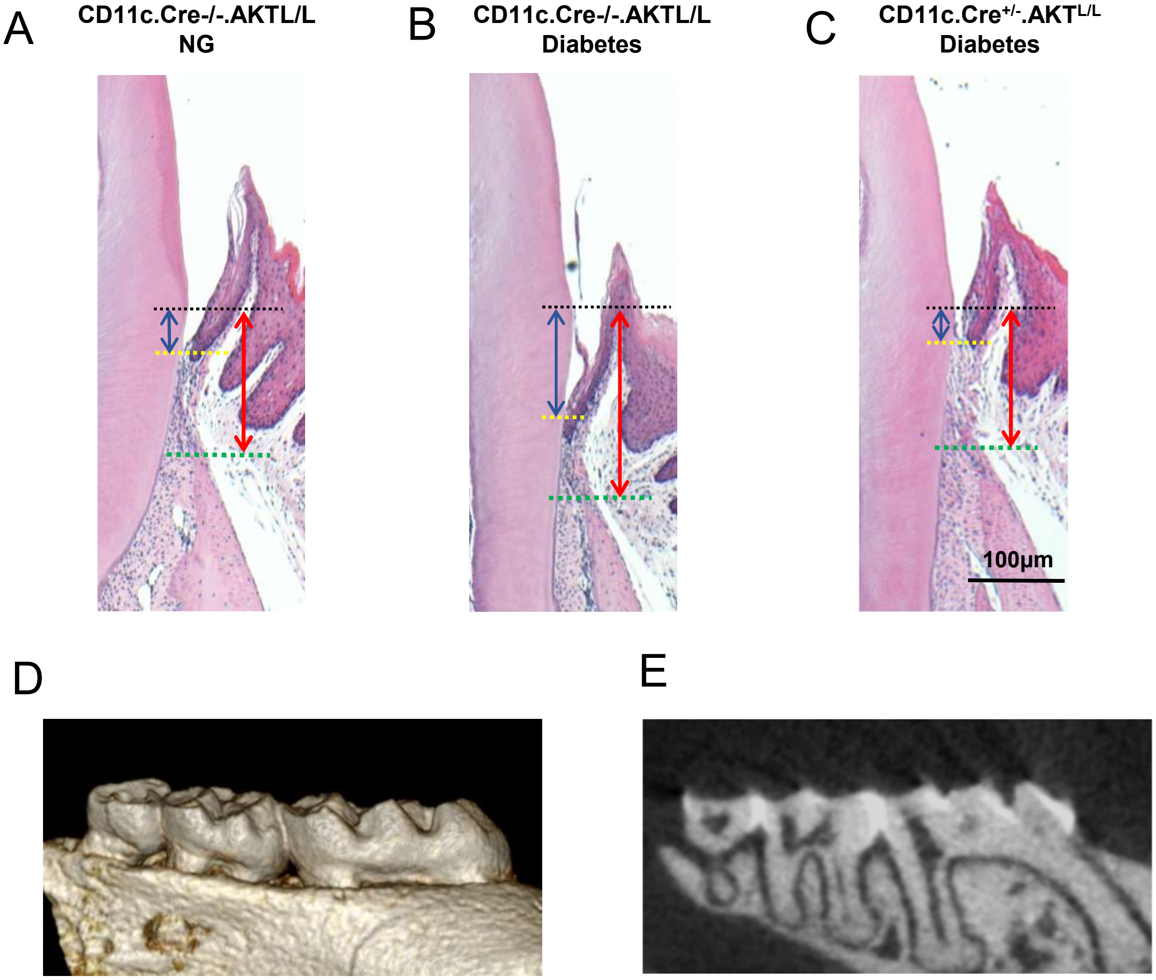

Figure S2： Flow cytometry and FMO for the CD11c and CD137L expression in mouse gingival cells.

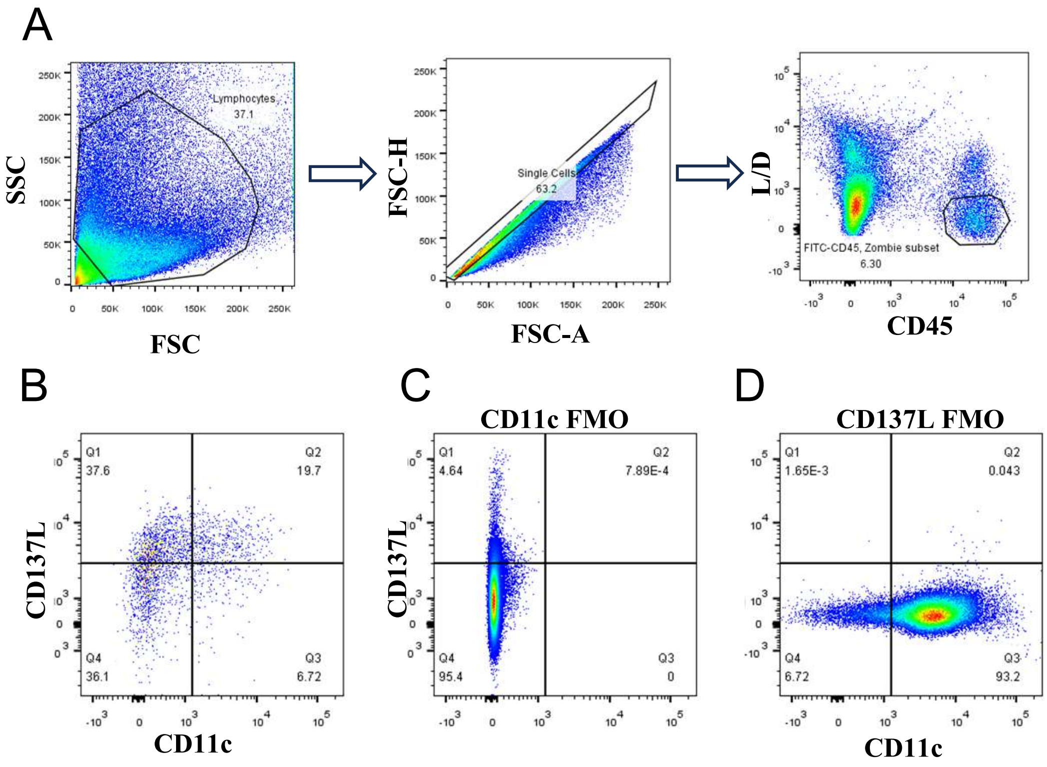

Figure S3: Flow cytometry of cells isolated from murine gingiva examined for γδ TCR, CD137 and Ki67.

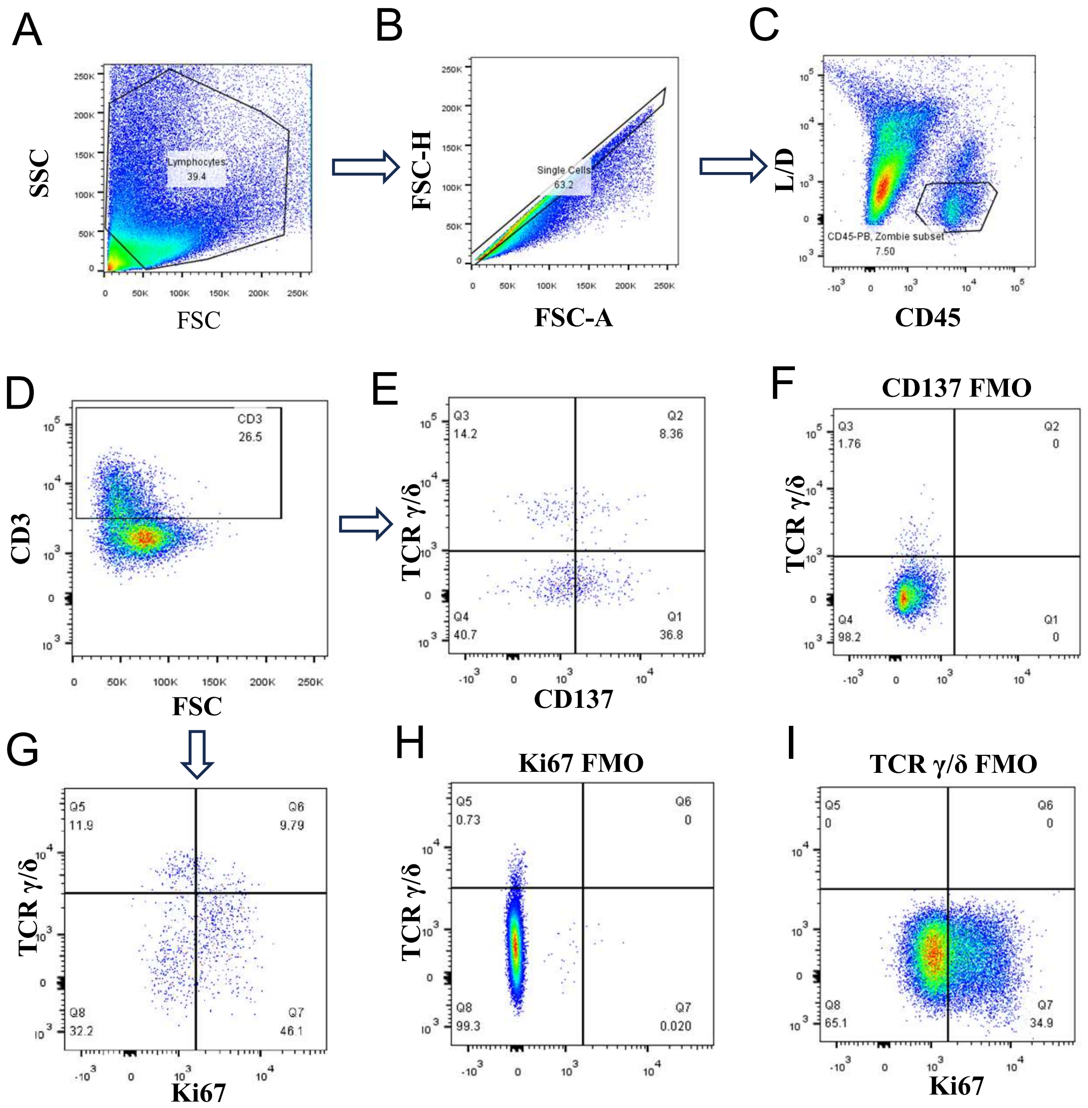

Figure S4: Flow cytometry of cells isolated from murine gingiva examined for Foxp3, Ki67 and CD137 expression.

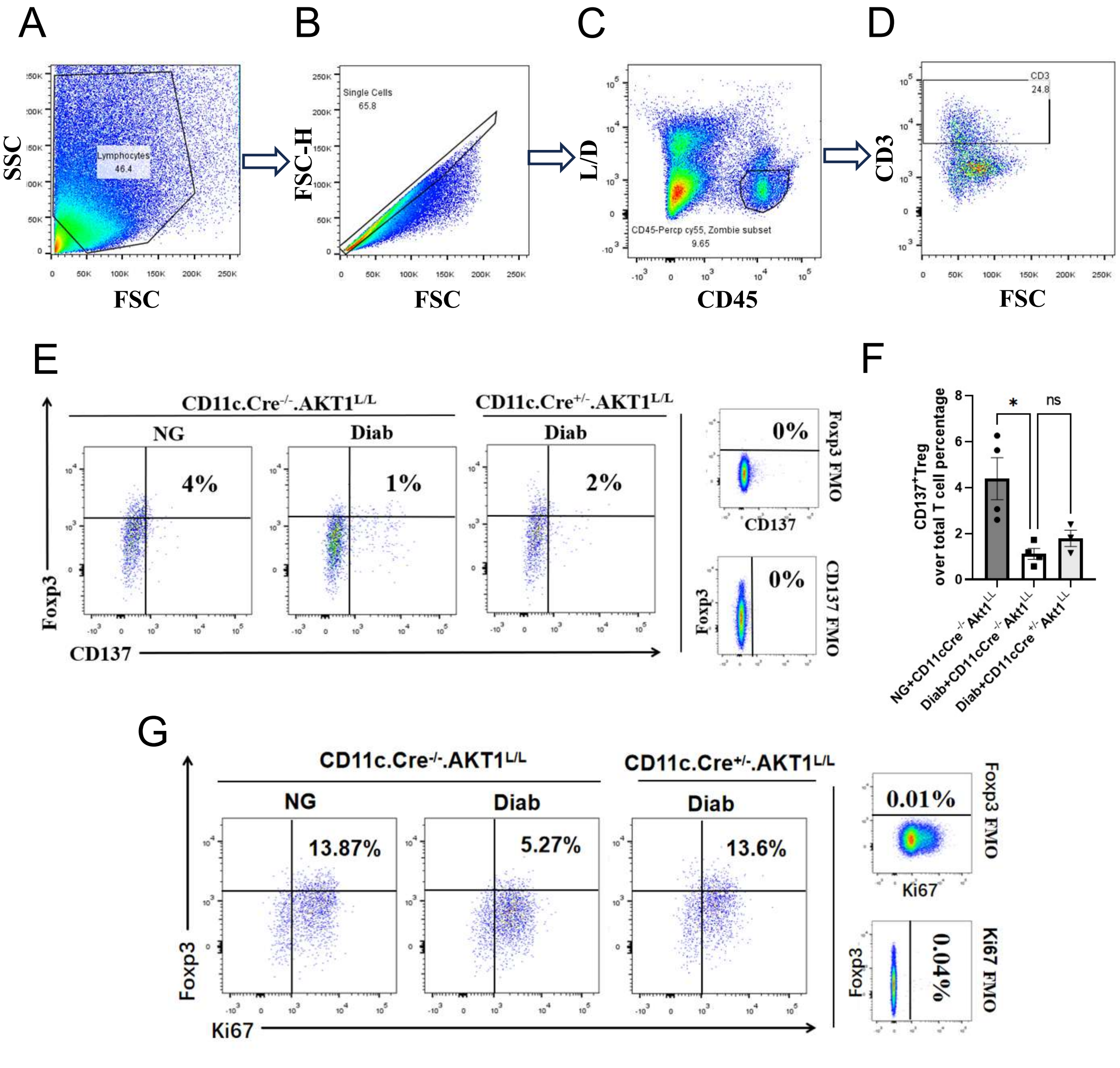

Figure S5: Flow cytometry of γδ T-cells following incubation in co-cultures with DC.

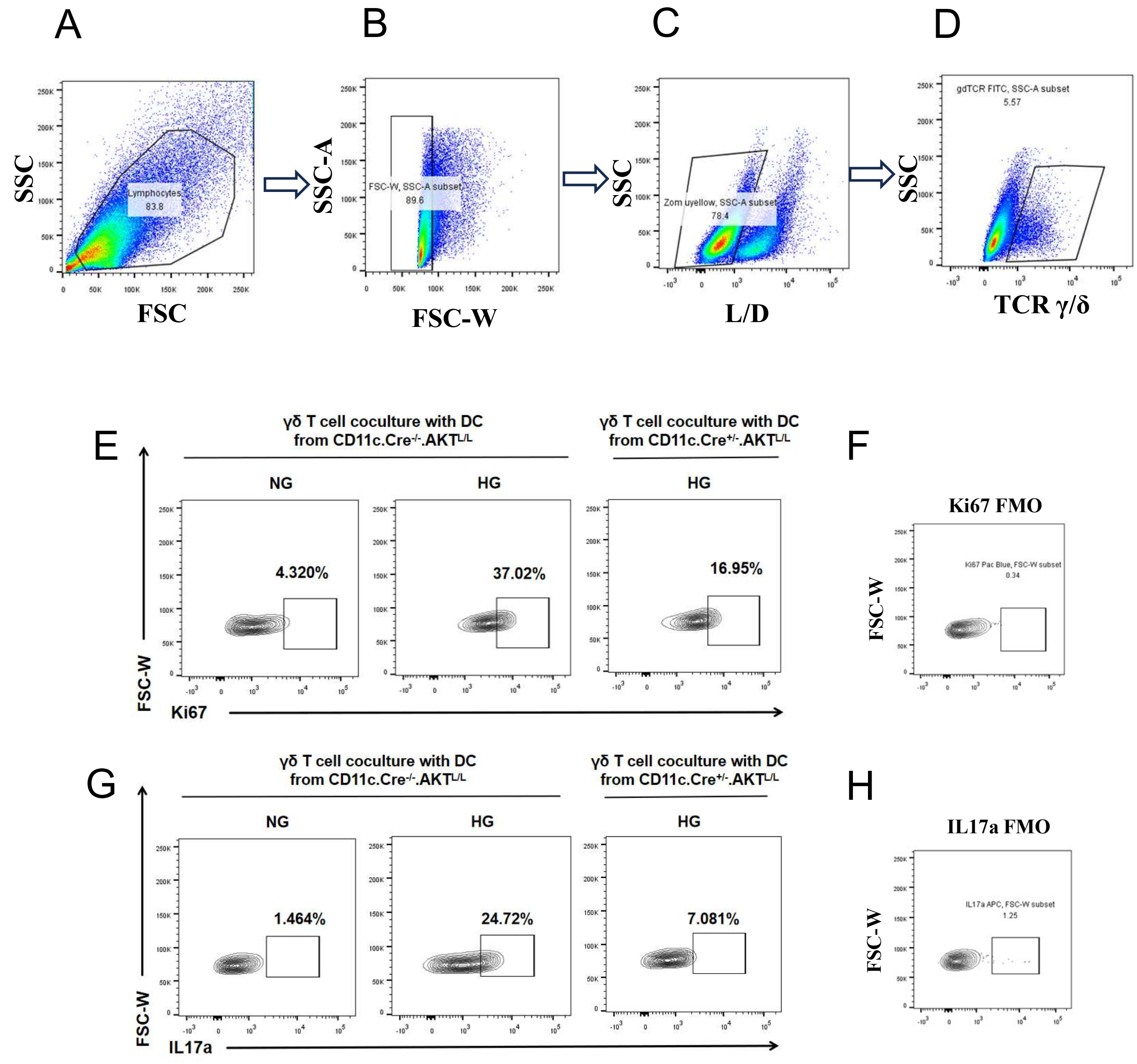

Figure S6: Flow cytometry of cells isolated from human gingiva and examined by flow cytometry for expression of HLA-DR, CD11c and CD137L.

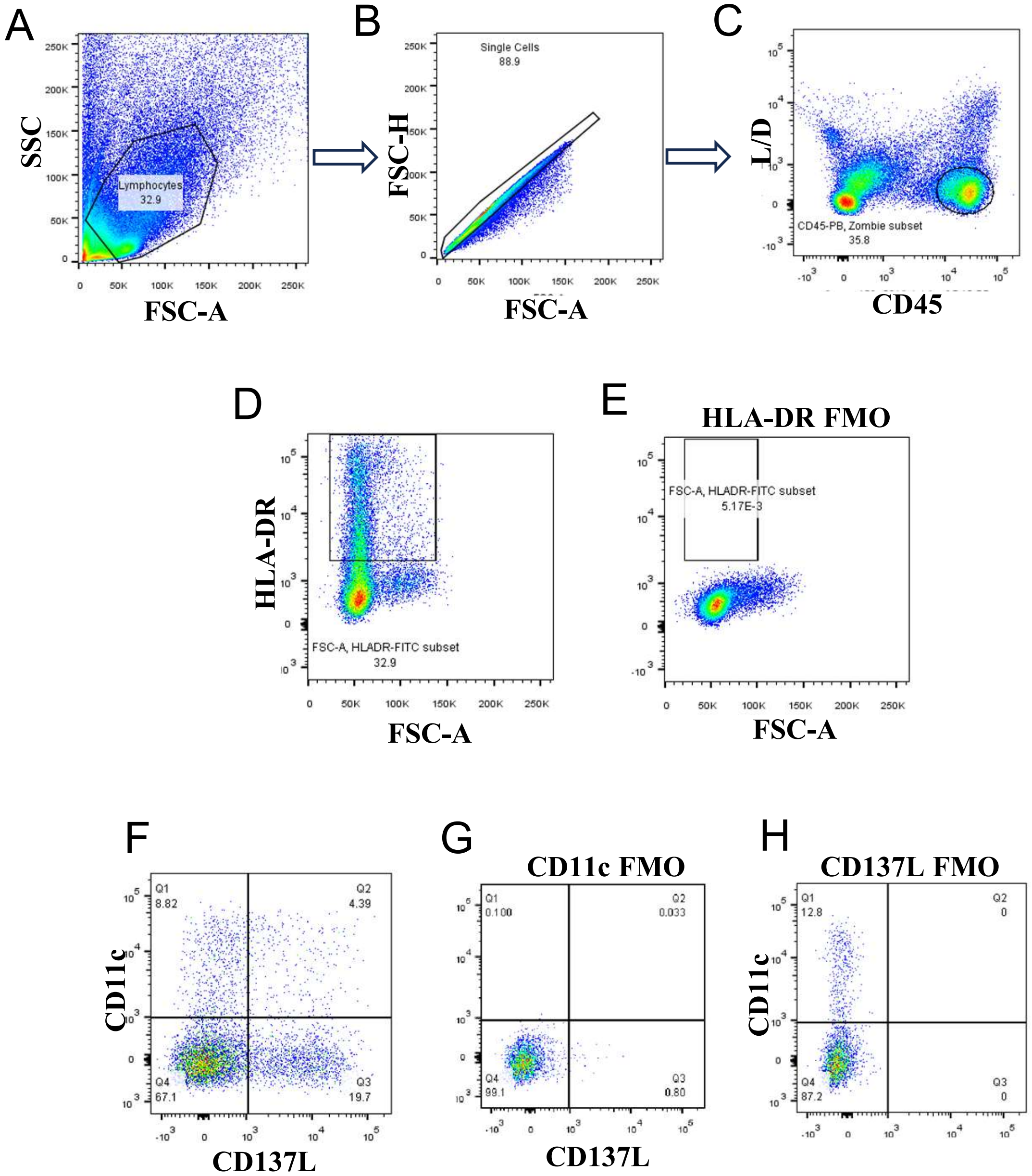

Figure S7: Flow cytometry of cells isolated from gingiva of diabetic mice injected with a CD137L antagonist or normoglycemic mice injected with a CD137 agonist.

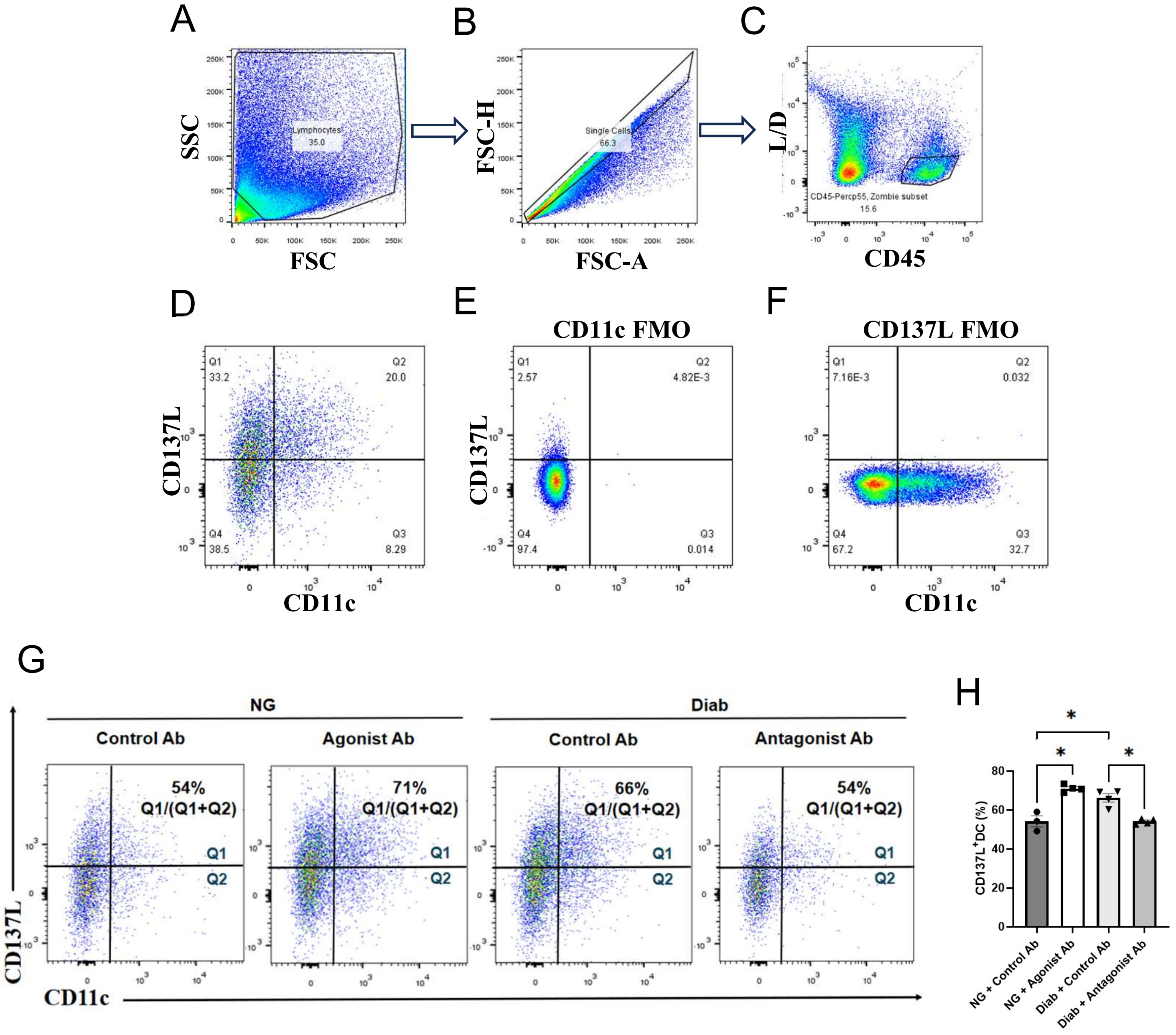

Figure S8: Flow cytometric detection of gingival neutrophils in CD11c.Cre$^{-/-}$.AKT1$^{L/L}$ and CD11c.Cre$^{+/-}$.AKT1$^{L/L}$ mice under normoglycemic and diabetic conditions.

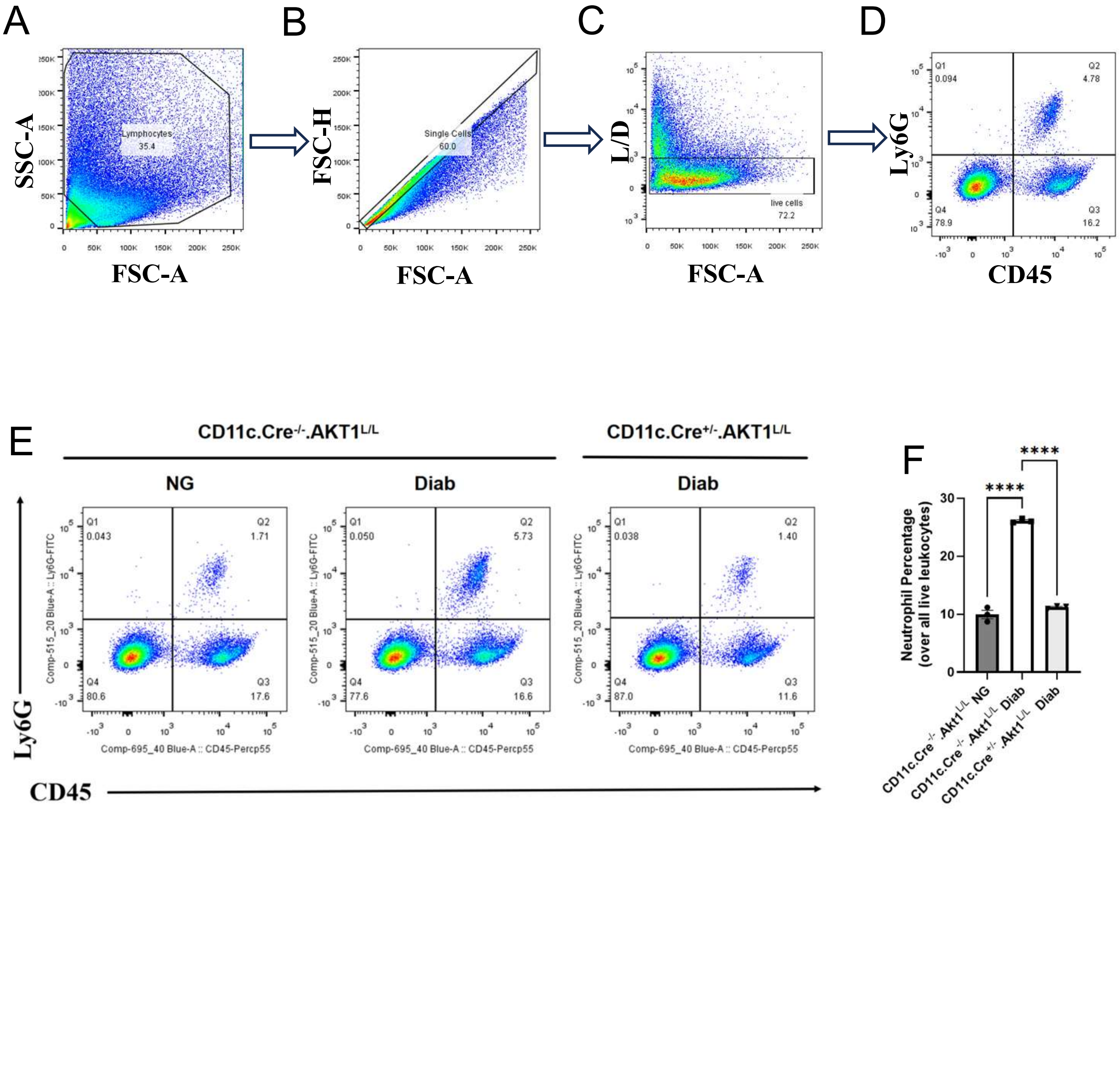

Figure S9: Flow cytometry of cells isolated from murine gingiva examined for γδ T-cells.

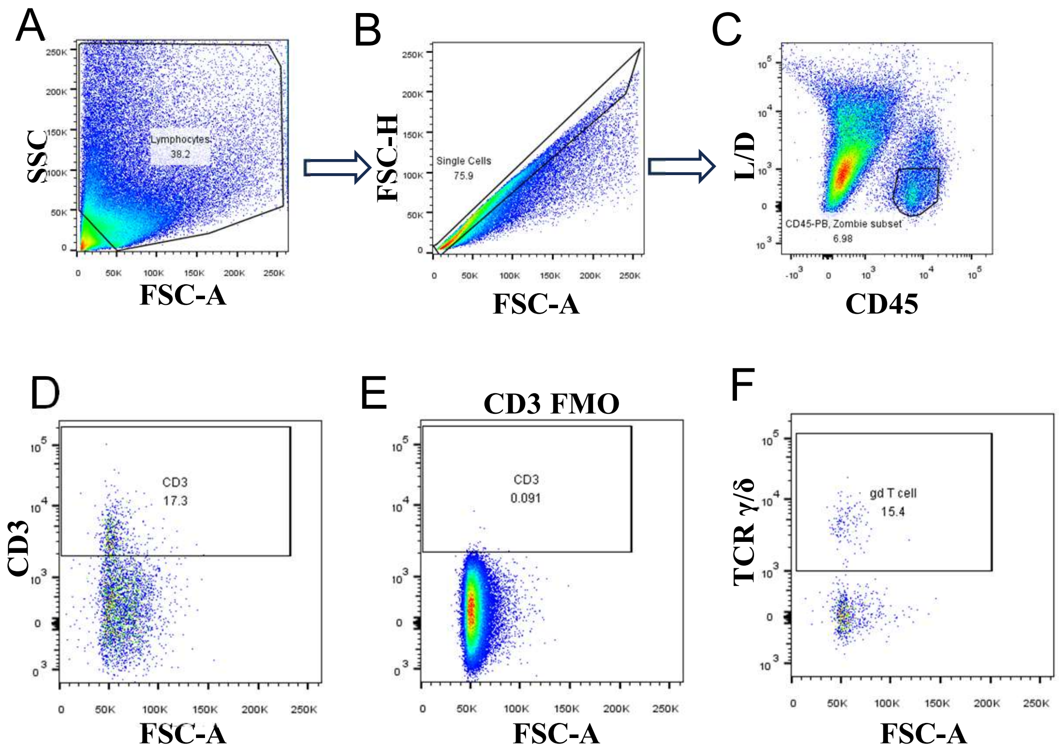

Supplemental Figure Legends:

Figure S1: Histologic and microCT analyses of periodontal tissues.
Periodontitis was induced and tissue obtained as described in Figure 1. (A-C) Representative images of H&E-stained images from the CD11c.Cre$^{-/-}$.AKT$^{L/L}$ normoglycemic (NG) (A), CD11c.Cre$^{-/-}$.AKT$^{L/L}$ diabetes (B), and CD11c.Cre$^{+/-}$.AKT$^{L/L}$ diabetes groups (C). The dotted lines represent the following: Black, cementoenamel junction; yellow, epithelial attachment; green, coronal aspect of the alveolar bone crest. Vertical red arrows show the distance from the cementoenamel junction to the alveolar bone crest, which indicate relative bone loss; blue arrows show the distance from the cementoenamel junction to the base of the epithelial attachment, which indicate periodontal attachment loss. (D) Representative three-dimensional (3D) microCT images of the maxillary molar region in normoglycemic control mice that were inoculated with vehicle alone instead of bacteria. (E) Corresponding sagittal section through the reconstructed 3D image in panel D showing normal bone height and periodontal tissue contour, serving as a baseline reference for quantitative comparison with diabetic and experimental periodontitis groups.

Figure S2：Flow cytometry and FMO for the CD11c and CD137L expression in mouse gingival cells.
Periodontitis was induced and cells were isolated from gingival tissue as described in Figure 1A. (A) Initial gating by flow cytometry was performed to exclude debris and to identify cells based on cell size (forward scatter, FSC) and granularity (side scatter, SSC). Cells with low FSC and SSC values were gated out. Single cell populations were identified by plotting FSC-A (area) vs. FSC-H (height) to exclude doublets. Only singlet cells were included in subsequent analysis. The viable leukocytes were identified as Zombie^{-}CD45^{+}. (B) The CD137L^{+} DCs were identified as CD137L^{+}CD11c^{+} cells. (C-D) FMO controls for CD11c (C) and CD137L (D).

Figure S3: Flow cytometry of cells isolated from murine gingiva examined for γδ TCR, CD137 and Ki67.
Periodontitis was induced and flow cytometry was carried out on cells isolated from murine gingival tissue as described in Figure 1A. (A) Forward scatter (FSC) vs. side scatter (SSC) flow cytometry plot. Initial gating was performed to exclude debris and to identify the main cell population based on cell size and granularity. Cells with low FSC and SSC values were gated out. (B) Single cells were identified by plotting FSC-A (area) vs. FSC-H (height) to exclude doublets. Only singlet cells were included in subsequent analysis. (C) Viable leukocytes were identified as Zombie-CD45^{+}. (D) T cells were identified as CD3^{+} population. (E)The CD137^{+}γδ T cells were gating from the CD137^{+}γδ TCR^{+} population. (F) Fluorescence Minus One (FMO) Controls for CD137. (G)The Ki67^{+}γδ T cells were gating from the Ki67^{+}γδ TCR^{+} population. (H-I) Fluorescence minus one (FMO) Controls for Ki67(H) and γδ TCR(I).

Figure S4: Flow cytometry of cells isolated from murine gingiva examined for Foxp3, Ki67 and CD137 expression. Periodontitis was induced and flow cytometry was carried out on cells isolated from murine gingival tissue as described in Figure 1A. (A) Initial gating was performed to exclude debris and to identify the primary cell population based on cell size and granularity. Cells with low FSC and SSC values were gated out. (B) Single cells were identified by plotting

FSC-A (area) vs. FSC-H (height) to exclude doublets. Only singlet cells were included in subsequent analysis. (C) Viable leukocytes were identified as $Zombie^{-}CD45^{+}$. (D) T cells were identified as $CD3^{+}$ population. (E) Representative flow cytometry plots of the $CD137^{+}$ $Foxp3^{+}$ Treg population and FMO controls for Foxp3 (top) and CD137 (bottom). (F) Quantification of proliferating $Foxp3^{+}$ $CD137^{+}$ Tregs normalized by total $CD3^{+}$ T cell count across three mouse groups. (G) Representative flow cytometry plots of the $Ki67^{+}$ $Foxp3^{+}$ Treg population and FMO controls for Foxp3 (top) and Ki67 (bottom). For panels F: N = 3-4 biologic replicates per group. One-way ANOVA with Tukey's post hoc test, *$p<0.05$, ns: no significance.

Figure S5: Flow cytometry of γδ T-cells following incubation in co-cultures with DC. Dendritic cells and γδ T-cells were co-cultured in vitro in media with normal glucose levels (NG) or 25mM high glucose (HG) and then examined by flow cytometry. (A) Initial gating was performed to exclude debris and to identify cells based on size and granularity. Cells with low FSC and SSC values were gated out. (B) Single cell populations were identified by plotting FSC-A (area) vs. FSC-H (height) to exclude doublets. Only singlet cells were included in subsequent analysis. (C) Viable cells were identified as $Zombie^{-}$. (D) γδ T cells were identified as γδ TCR^{+} population. (E, F) Representative flow cytometry plots of Ki67 expression in γδ T cells co-cultured with DC from different genotypes under normoglycemic (NG) or high glucose (HG) conditions. FMO control dot plots are shown. (G, H) Representative flow cytometry plots IL-17a expression in γδ T cells co-cultured with DCs from different genotypes under normoglycemic (NG) or high glucose (HG) conditions. FMO control dot plots.

Figure S6: Flow cytometry of cells isolated from human gingiva and examined by flow cytometry for expression of HLA-DR, CD11c and CD137L.

(A) Initial gating was performed to exclude debris and to identify cells based on size and granularity. Cells with low FSC and SSC values were gated out. (B) Single cells were identified by plotting FSC-A (area) vs. FSC-H (height) to exclude doublets. Only singlet cells were included in subsequent analysis. (C) Viable cells were identified as $Zombie^{-}CD45^{+}$. (D) The HLA-DR^{+} leukocytes were gated using an HLA-DR antibody. (E) FMO control for HLA-DR. (F) Identification of $CD11c^{+}$ and $CD137L^{+}$ cells. (G-H) FMO controls for CD11c and CD137L.

Figure S7: Flow cytometry of cells isolated from gingiva of diabetic mice injected with a CD137L antagonist or normoglycemic mice injected with a CD137 agonist.

Diabetic mice were injected with a CD137L antagonist and normoglycemic mice were injected with a CD137 agonist. Periodontitis was induced as described in Figure 1. (A) Initial gating was performed to exclude debris and to identify cell populations based on cell size and granularity. Cells with low FSC and SSC values were gated out. (B) Single cells were identified by plotting FSC-A (area) vs. FSC-H (height) to exclude doublets. Only singlet cells were included in subsequent analysis. (C) Viable cells were identified as $Zombie^{-}$. (D) Representative scatter plots for detection of $CD137L^{+}$ and $CD11c^{+}$ cells. (E-F) FMO control for CD11c and CD137L, respectively. (G) Scatter plots of $CD137L^{+}$ and $CD11c^{+}$ populations in normoglycemic mice that received control antibody (NG+Ab), normoglycemic mice that received CD137 agonist antibody (NG+Agonist Ab), diabetic mice that received control antibody (Diab+Control Ab) and diabetic mice that received CD137L antagonist antibody (Diab+Antagonist Ab). (H) Quantitative assessment of $CD137L^{+}CD11c^{+}$ double positive

cells. N=3-4 biologic replicates per group, one-way ANOVA followed by post hoc test. Statistical significance was determined at *$p<0.05$.

Figure S8: Flow cytometric detection of gingival neutrophils in CD11c.Cre-/-.AKT1L/L and CD11c.Cre+/-.AKT1L/L mice under normoglycemic and diabetic conditions.

(A) Initial gating was performed to exclude debris and to identify cell populations based on cell size and granularity. Cells with low FSC and SSC values were gated out. (B) Single cells were identified by plotting FSC-A (area) vs. FSC-H (height) to exclude doublets. Only singlet cells were included in subsequent analysis. (C) Viable cells were identified as Zombie^{-}. (D) Representative scatter plots for detection of CD45^{+} and Ly6G^{+} neutrophils. (E) Representative neutrophil (CD45+Ly6G+) gating in urine gingival cells from CD11c.Cre$^{-/-}$.AKT$^{L/L}$ NG, CD11c.Cre$^{-/-}$.AKT$^{L/L}$ diabetes, and CD11c.Cre+/-.AKTL/L diabetes groups. (F) Quantification of neutrophil percentages normalized to live CD45+ cells across the three groups. N = 3 per each group, one-way ANOVA and Tukey's post hoc test; *$p<0.05$.

Figure S9: Flow cytometry of cells isolated from murine gingiva examined for γδ T-cells.

Periodontitis was induced as in Figure 1 and isolated cells from murine gingiva were examined by flow cytometry. (A) FSC-A and SSC gating. (B) Single cells were identified by plotting FSC-A (area) vs. FSC-H (height) to exclude doublets. Only singlet cells were included in subsequent analysis. (C) Viable CD45^{+} cells were identified as Zombie^{-}. (D) Gating parameters to identify CD3^{+} T cells. (E) FMO control for CD3. (F) Gating parameters for quantification of TCRγδ^{+} cells.

Supplemental Table S1 The Quality Control Assessment of scRNA-seq Transcripts

Group	Number of cells	Mean reads/cell	Reads mapped to genome	Saturation	Total genes detected
NG+CD11c.Cre-/-.AKT1L/L	11124	50630	95%	76%	20627
Diab+CD11c.Cre-/-.AKT1L/L	10496	45576	94%	74%	21757
Diab+CD11c.Cre+/-.AKT1L/L	10944	55640	95%	76%	22489

Table S1: Single-cell RNA sequencing (scRNA-seq) was carried out to examine the transcription profile of cells of the mouse gingiva tissue following induction of periodontitis by oral inoculation of P gingivalis and F nucleatum in three distinct experimental groups: normoglycemic CD11c.Cre-/-.AKTL/L, diabetic CD11c.Cre-/-.AKTL/L, and diabetic CD11c.Cre+/-.AKTL/L. The quality control assessment of scRNA-seq transcripts was presented in the table.

Supplemental Table S2 Key Resources Table

REAGENT or RESOURCE	SOURCE	IDENTIFIER	Clone
Antibodies			
PerCP/Cyanine5.5 anti-mouse CD45	Biolegend	Cat#103131; RRID:AB_893340	30-F11
Pacific Blue™ anti-mouse CD45	Biolegend	Cat#103126; RRID:AB_493535	30-F11
PE anti-mouse CD137	Biolegend	Cat#106105; RRID:AB_2287565	17B5
Brilliant Violet 421™ anti-mouse F4/80	Biolegend	Cat#123131; RRID:AB_10901171	BM8
PE/Cyanine7 anti-mouse CD11c	Biolegend	Cat#117317; RRID:AB_493568	N418
APC/Cyanine7 anti-mouse CD11c	Biolegend	Cat#117323; RRID:AB_830646	N418
FITC anti-mouse Ly6G	Biolegend	Cat#127606; RRID:AB_1236488	1A8
Brilliant Violet 650™ anti-mouse CD3	Biolegend	Cat#100229; RRID:AB_11204249	17A2
APC/Cyanine7 anti-mouse TCR γ/δ	Biolegend	Cat#118143; RRID:AB_2892275	GL3
FITC anti-mouse TCR γ/δ	Biolegend	Cat#107503; RRID:AB_313312	UC7-13D5
PE/Cyanine7 anti-mouse Ki67	Biolegend	Cat#151217; RRID:AB_2910305	11F6
Brilliant Violet 421™ anti-mouse Ki67	Biolegend	Cat#652411; RRID:AB_2562663	16A8
APC anti-mouse IL-17A	Biolegend	Cat#506915; RRID:AB_536017	TC11-18H10.1
anti-mouse IL-17A	Proteintech Group	Cat#26163-1-AP; AB_2880409	N/A
APC anti-mouse Foxp3	Invitrogen	Cat#17-5773-82	FJK-16s
Zombie Yellow™ Fixable Viability Kit	Biolegend	Cat#423104	RUO
Alexa Fluor® 647 AffiniPure® Donkey Anti-Rabbit IgG	Jackson ImmunoResearch	Cat#711-605-152	N/A
Rabbit IgG	Vector Laboratories	Cat#I-1000-5	N/A
Purified anti-mouse CD16/32 Antibody	Biolegend	Cat#101302	93

Pacific Blue™ anti-human CD45 Antibody	Biolegend	Cat#304021	HI30
FITC anti-human HLA-DR Antibody	Biolegend	Cat#307603	L243
APC anti-human CD11c Antibody	Biolegend	Cat#337207	Bu15
PE anti-human CD137L (4-1BB Ligand) Antibody	Biolegend	Cat#311503	5F4
Human TruStain FcX™ (Fc Receptor Blocking Solution)	Biolegend	Cat#422301	N/A

REAGENT or RESOURCE	SOURCE	IDENTIFIER
Bacterial and virus strains		
Porphyromonas gingivalis	ATCC	CAT#33277
Fusobacterium nucleatum	ATCC	CAT#25586
Chemicals, peptides, and recombinant proteins		
DNAseI	Sigma-Aldrich	Cat#10104159001
Collagenase IV	Worthington	Cat#LS004188
Dispase II	Sigma-Aldrich	Cat#D4693-1G
ACK lysis buffer	Biolegend	Cat#420301
DAPI	Abcam	Cat#ab104139
FOXP3 Perm Buffer (10x)	Biolegend	Cat#421402
Flow Cytometry Fix Buffer	Proteintech	Cat#PF00016
Critical commercial assays		
PrimeScript RT reagent Kit	Takara	Cat# RR037A
Experimental models: Organisms/strains		
Mouse: CD11c.Cre	Jackson Laboratory	Stock # 8068; B6; Bar Habor, ME, USA
Mouse: Akt1fl/fl	Dr. Morris Birnbaum' lab.	N/A
Oligonucleotides		
Primers for PCR, see Table S1	BGI Genomics	N/A
Software and algorithms		
GraphPad Prism v8.3	GraphPad Software	www.graphpad.com
FlowJo v10	Tree Star	N/A
NIS Elements [AR 5.20.02 64-bit]	Nikon Instruments Inc	N/A

Supplemental Table S3 Comparison of *Cd137l* Differential Expression Analysis in Monocytes Subcluster

	Subcluster	Compare	avg_log2FC	pct.1	pct.2	p_val	p_val_adj
Cd137l	6 (DC)	**Diab VS. NG**	**1.42**	**0.86**	**0.76**	**0.00**	**0.00**
		**AKT KO Diab VS. Diab**	**-1.06**	**0.79**	**0.86**	**0.00**	**0.00**
	7 (DC)	**Diab VS. NG**	**0.92**	**0.74**	**0.59**	**0.00**	**0.01**
		**AKT KO Diab VS. Diab**	**-0.72**	**0.58**	**0.74**	**0.00**	**0.01**
	19 (DC)	Diab VS. NG	1.35	0.73	0.50	0.01	1.00
		AKT KO Diab VS. Diab	-0.82	0.72	0.73	0.16	1.00
	2 (Macrophage)	**Diab VS. NG**	**0.91**	**0.61**	**0.54**	**0.00**	**0.00**
		AKT KO Diab VS. Diab	-0.33	0.67	0.61	0.02	0.26

Table S3: Comparison of *Cd137l* expression in monocyte subclusters between diabetic and normoglycemic mice or Akt1 KO diabetic and diabetic. Cluster 6 and 7 (DCs) were the only clusters in which *Cd137l* expression was elevated significantly in the diabetic group compared to the normoglycemic control and reversed by Akt1 KO. Bold values indicate markers with an adjusted p-value < 0.05. All p-values were adjusted for multiple testing using the Benjamini-Hochberg method.

Supplemental Table S4: Fold Changes of IL17A+/CD3+ Cells Post Bacterial Infections in mice

	2 weeks	6 weeks
Normoglycemic	1.86 ±0.25	1.89 ±0.31
Diabetic	2.68 ±0.50*	3.21 ±0.15*

Table S4: The induction of periodontitis following bacterial inoculation stimulated IL17A expression compared to baseline in lymphocytes as determined by flow cytometry for both Normoglycemic and Diabeitc mice groups over a 2-6 week time period, the IL17A+/CD3+ percentage in Diabetic mice was significantly greater. *: $P<0.05$ between groups
